# Longitudinal Assessment of Mental Health Disorders and Comorbidities Across 4 Decades Among Participants in the Dunedin Birth Cohort Study

**DOI:** 10.1001/jamanetworkopen.2020.3221

**Published:** 2020-04-21

**Authors:** Avshalom Caspi, Renate M. Houts, Antony Ambler, Andrea Danese, Maxwell L. Elliott, Ahmad Hariri, HonaLee Harrington, Sean Hogan, Richie Poulton, Sandhya Ramrakha, Line J. Hartmann Rasmussen, Aaron Reuben, Leah Richmond-Rakerd, Karen Sugden, Jasmin Wertz, Benjamin S. Williams, Terrie E. Moffitt

**Affiliations:** 1 Department of Psychology and Neuroscience, Duke University, Durham, North Carolina; 2 Department of Psychiatry and Behavioral Sciences, Duke University School of Medicine, Durham, North Carolina; 3 Center for Genomic and Computational Biology, Duke University, Durham, North Carolina; 4 Social, Genetic, and Developmental Psychiatry Research Centre, Institute of Psychiatry, Psychology, and Neuroscience, King’s College London, London, United Kingdom; 5 PROMENTA Center, University of Oslo, Oslo, Norway; 6 Dunedin Multidisciplinary Health and Development Research Unit, University of Otago, Dunedin, New Zealand; 7 Clinical Research Centre, Copenhagen University Hospital Amager and Hvidovre, Copenhagen, Denmark; 8 Center for Developmental Science, University of North Carolina at Chapel Hill, Chapel Hill

## Abstract

**Importance:**

Mental health professionals typically encounter patients at 1 point in patients’ lives. This cross-sectional window understandably fosters focus on the current presenting diagnosis. Research programs, treatment protocols, specialist clinics, and specialist journals are oriented to presenting diagnoses, on the assumption that diagnosis informs about causes and prognosis. This study tests an alternative hypothesis: people with mental disorders experience many different kinds of disorders across diagnostic families, when followed for 4 decades.

**Objective:**

To describe mental disorder life histories across the first half of the life course.

**Design, Setting, and Participants:**

This cohort study involved participants born in New Zealand from 1972 to 1973 who were enrolled in the population-representative Dunedin Study. Participants were observed from birth to age 45 years (until April 2019). Data were analyzed from May 2019 to January 2020.

**Main Outcomes and Measures:**

Diagnosed impairing disorders were assessed 9 times from ages 11 to 45 years. Brain function was assessed through neurocognitive examinations conducted at age 3 years, neuropsychological testing during childhood and adulthood, and midlife neuroimaging-based brain age.

**Results:**

Of 1037 original participants (535 male [51.6%]), 1013 had mental health data available. The proportions of participants meeting the criteria for a mental disorder were as follows: 35% (346 of 975) at ages 11 to 15 years, 50% (473 of 941) at age 18 years, 51% (489 of 961) at age 21 years, 48% (472 of 977) at age 26 years, 46% (444 of 969) at age 32 years, 45% (429 of 955) at age 38 years, and 44% (407 of 927) at age 45 years. The onset of the disorder occurred by adolescence for 59% of participants (600 of 1013), eventually affecting 86% of the cohort (869 of 1013) by midlife. By age 45 years, 85% of participants (737 of 869) with a disorder had accumulated comorbid diagnoses. Participants with adolescent-onset disorders subsequently presented with disorders at more past-year assessments (*r* = 0.71; 95% CI, 0.68 to 0.74; *P* < .001) and met the criteria for more diverse disorders (*r* = 0.64; 95% CI, 0.60 to 0.67; *P* < .001). Confirmatory factor analysis summarizing mental disorder life histories across 4 decades identified a general factor of psychopathology, the p-factor. Longitudinal analyses showed that high p-factor scores (indicating extensive mental disorder life histories) were antedated by poor neurocognitive functioning at age 3 years (*r* = −0.18; 95% CI, −0.24 to −0.12; *P* < .001), were accompanied by childhood-to-adulthood cognitive decline (*r* = −0.11; 95% CI, −0.17 to −0.04; *P* < .001), and were associated with older brain age at midlife (*r* = 0.14; 95% CI, 0.07 to 0.20; *P* < .001).

**Conclusions and Relevance:**

These findings suggest that mental disorder life histories shift among different successive disorders. Data from the present study, alongside nationwide data from Danish health registers, inform a life-course perspective on mental disorders. This perspective cautions against overreliance on diagnosis-specific research and clinical protocols.

## Key Points

**Question:**

Do mental disorder life histories shift among different successive disorders?

**Findings:**

In this cohort study of 1037 participants in the Dunedin Study birth cohort, with assessments from ages 11 to 45 years, mental disorder life histories shifted among different successive internalizing, externalizing, and thought disorders. Mental disorder life histories are better described by age of onset, duration, and diversity of disorder than by any particular diagnosis.

**Meaning:**

The finding that most mental disorder life histories involve different successive disorders helps to account for genetic and neuroimaging findings pointing to transdiagnostic causes and cautions against overreliance on diagnosis-specific research and clinical protocols.

## Teaser

This cohort study describes mental disorder life histories across 4 decades among participants in the Dunedin Study.

## Introduction

The practice of diagnosing mental disorders is at a crossroads. The *Diagnostic and Statistical Manual of Mental Disorders*, which guides diagnostic practice, is being questioned.^1^ The US National Institute of Mental Health has called for a new approach to studying mental disorders,^2^ and the public is confused about what constitutes a mental disorder, resulting in a practice known as diagnosis shopping.^3^ Our thesis is that progress in conceptualizing mental disorders has been delayed by the field’s limiting focus on cross-sectional information. This study demonstrates how much novel information can be learned by taking a longitudinal, life-course view of mental disorders.

Researchers and clinicians in mental health fields typically encounter a patient at 1 point in the patient’s life, and, accordingly, tend to study or treat the disorders that can be diagnosed at that time. This short-term view promotes the idea that patients can be adequately characterized by their current presenting diagnoses. Research hypotheses and clinical protocols tend to be tailored to diagnoses, resulting in diagnosis-specific therapies, clinics, journals, and professional societies, and even diagnosis-specific funding agencies. Such tailoring is based on the assumption that a diagnosis provides information about the causes of the patient’s disorder and that tailoring treatment to a diagnosis will ensure a good response and prognosis. However, the wisdom of overemphasizing a diagnosis is challenged by new evidence from neuroimaging studies,^4,5,6,7,8^ genetic studies,^9,10,11^ and risk-prediction studies,^12,13,14^ which reveal that major etiological findings are transdiagnostic. Moreover, since publication of the *Diagnostic and Statistical Manual of Mental Disorders* (Third Edition) (*DSM-III*),^15^ evidence has accumulated that sets of disorders and symptoms predictably co-occur.^16,17^ Depression and anxiety disorders (ie, the internalizing family) emerge in the same patient, disruptive disorders and substance abuse (ie, the externalizing family) emerge in the same patient, and disorganized thoughts, delusional beliefs, hallucinations, obsessions and compulsions (ie, the thought disorder family) emerge in the same patient. As a result of such empirical studies about the structure of psychopathology, these disorder families are now accommodated in research,^18^ and transdiagnostic treatments are increasing in popularity.^19^

Of note, most research on the structure of psychopathology has been conducted using data collected at 1 time point, but one must consider the following questions: what if most patients tend to meet the criteria for many different diagnoses in turn, not only within 1 diagnostic family, but across families, too? What if the predominant pattern were one in which the onset of mental disorder occurs in the first decades of life and, thereafter, whenever an individual is assessed for a disorder, that individual might meet the criteria for a succession of different diagnoses? These questions are of pragmatic significance because much of the work of mental health professionals is driven by cross-sectionally assessed diagnoses.

One remarkable study^20^ confirmed that most patients do meet the criteria for many different diagnoses in turn. In that study,^20^ every mental disorder diagnosed was associated with an increased risk that the patient would be diagnosed at another time with other disorders, both inside and outside the index disorder’s family. Using Danish registers of inpatient and outpatient clinics, the study covered nearly 2 decades and included nearly 6 million individuals. Nevertheless, the Berkson bias^21^ could exaggerate the picture of comorbidity in these registers, because greater comorbidity and duration of impairment are associated with a greater likelihood of treatment. Patients in clinical registers typically have unusually complex cases and many comorbid disorders lasting many years.^21,22^ Registers omit patients treated in primary care and also the many community dwellers whose disorder goes untreated. Thus, it is possible that crossing diagnostic families is unique to clinical patients but does not generalize to the fuller population of individuals experiencing mental disorder. Another potential artifact in clinical registers is the possibility of inconsistent diagnostic practices by a series of clinicians seeing the same patient at different times. Here we report a replication and extension of research begun in Danish registers, using a population-representative birth cohort whose mental health has been tracked regardless of treatment status and repeatedly assessed in a systematic, standardized manner for 4 decades.

The cohort that we tracked, the Dunedin Study, is unique in the annals of psychiatric epidemiology. In 1983 and 1984, when participants were aged 11 years, it was the first cohort to measure disorders in children using standardized diagnostic interviews.^23^ Research diagnoses have been made on 9 occasions with strong participant retention, until participants turned age 45 years. This diagnostic time-series allowed us to describe mental disorder life histories in terms of 3 developmental parameters: age of onset, duration, and comorbid diversity among disorder families. We then applied confirmatory factor analysis to symptoms to summarize participants’ mental disorder life histories with a general factor of psychopathology, the p-factor, which has been previously described and replicated.^24,25^ We tested the hypothesis that mental disorder life histories, summarized by the p-factor, reflect compromised brain function, by examining associations with neurocognitive deficits at age 3 years, subsequent cognitive decline from childhood to adulthood, and advanced brain age in midlife, as derived from neuroimaging.

## Methods

### Sample

Participants were members of the Dunedin Study, a longitudinal investigation of a population-representative birth cohort (eAppendix 1 in the Supplement). The participants were all individuals born between April 1972 and March 1973 in Dunedin, New Zealand, who participated in the first assessment at age 3 years,^26^ representing 91% of participants who were eligible on the basis of residence in the province. The cohort represented the range of socioeconomic status on New Zealand’s South Island and in adulthood matched the New Zealand National Health and Nutrition Survey on key health indicators (eg, body mass index, smoking, and physician visits) and same-age citizens in the New Zealand Census on educational attainment.^26,27^ The cohort is primarily white (964 participants [93%]), matching South Island demographics. Assessments were held at birth and at ages 3, 5, 7, 9, 11, 13, 15, 18, 21, 26, 32, 38, and, most recently, 45 years, when 938 of the 997 living cohort members (94%) took part (completed April 2019).

Participants gave written informed consent. Protocols were approved by the institutional ethical review boards of the participating universities. This study follows the Strengthening the Reporting of Observational Studies in Epidemiology (STROBE) reporting guideline.

### Assessing Psychopathology

Beginning at age 11 years, participants were interviewed about past-year symptoms of mental disorders (eAppendix 2 in the Supplement). Interviews were conducted by health professionals, not lay interviewers. Interviewers were kept blind to participants’ prior data. At ages 11, 13, and 15 years, interviews were performed with the *Diagnostic Interview Schedule for Children*,^28^ assessing the following disorders: externalizing disorders (ie, attention-deficit/hyperactivity disorder and conduct disorder) and internalizing disorders (ie, depression, anxiety, and fears [including separation anxiety, overanxiety, social phobia, and simple phobia]). At ages 18, 21, 26, 32, 38, and 45 years, interviews were performed with the *Diagnostic Interview Schedule*,^29,30^ assessing the following disorders: externalizing disorders (ie, attention-deficit/hyperactivity disorder, conduct disorder, alcohol dependence, cannabis dependence, other drug dependence, and tobacco dependence), internalizing disorders (ie, depression, generalized anxiety disorder, fears [including social phobia, simple phobia, agoraphobia, and panic disorder], posttraumatic stress disorder, and eating disorders [including bulimia and anorexia]), and thought disorders (ie, obsessive-compulsive disorder, mania, and schizophrenia). As previously reported,^31^ a correlated-factor model showed that this 3-factor structure provided an excellent fit to the symptom-level data. Diagnoses, which followed the exclusionary criteria of various editions of the *Diagnostic and Statistical Manual of Mental Disorders*, were based on symptom algorithms and impairment ratings, but also incorporated information including standardized teacher, parent, and informant reports as developmentally appropriate; psychiatrists’ review of interviewers’ detailed case notes; pharmacists’ medication review; and staff ratings of symptoms observed.^32^ Up to age 15 years, diagnoses were made according to *DSM-III*^33^; at ages 18 and 21 years, diagnoses were made according to *Diagnostic and Statistical Manual of Mental Disorders* (Third Edition Revised)^34^; at ages 26, 32, and 38 years, diagnoses were made according to *Diagnostic and Statistical Manual of Mental Disorders* (Fourth Edition) (*DSM-IV*)^35^; and at age 45 years, diagnoses were made according to *Diagnostic and Statistical Manual of Mental Disorders* (Fifth Edition) (*DSM-5*),^36^ with the exception of substance-dependence disorders, which were diagnosed according to *DSM-IV* because *DSM-5* removed the dependence and abuse distinction. Review of treatment in the years between study assessments indicated that our net of 9 past-year diagnostic interviews captured all but 17 individuals treated in the 4 decades, most of whom had postpartum depression or were treated by a family doctor for anxiety or depression.

### Assessing Brain Function

Brain health at age 3 years, a composite measure, was derived from a 45-minute examination that included assessments by a pediatric neurologist; standardized tests of cognitive function, receptive language, and motor skills; and examiners’ ratings of emotional and behavioral regulation (eAppendix 3 in the Supplement). Cognitive function was measured at ages 7, 9, and 11 years using the Wechsler Intelligence Scale for Children–Revised^37^ and at age 45 years using the Wechsler Adult Intelligence Scale–IV.^38^ Cognitive decline was tested by estimating IQ at midlife after controlling for IQ in childhood.

Brain age at age 45 years was estimated using a publicly available algorithm^39^ that integrated structural neuroimaging measures of cortical thickness, cortical surface area, and subcortical volume to estimate the age of a person’s brain relative to their chronological age. T1-weighted structural magnetic resonance images were acquired using a 3-T scanner (Skyra; Siemens Healthcare) equipped with a 64-channel head-and-neck coil.

### Statistical Analysis

Raw visualization of diagnostic data was followed by cross-tabulations of mental disorders within and across time, calculating frequencies, percentages, and 95% CIs. Sankey diagrams were used to depict shifts in diagnosis across time. Confirmatory factor analysis was used to model the structure of psychopathology using symptom-level data (eAppendix 4 in the Supplement). Associations between variables were reported as sex-adjusted Pearson correlation coefficients (*r*) with 95% CIs. All 10 association tests (2-tailed *t* tests) reported were statistically significant after Bonferroni correction (*P* < .005). Data were analyzed using SAS statistical software version 9.4 (SAS Institute) and MPlus statistical software version 8.4 (Muthen & Muthen). Data were analyzed from May 2019 to January 2020.

## Results

### Longitudinal Patterns of Mental Disorder

Of 1037 original participants (535 male [51.6%]), 1013 had mental health data available. The following proportions of participants met the criteria for a mental disorder: 35% (346 of 975) at ages 11 to 15 years, 50% (473 of 941) at age 18 years, 51% (489 of 961) at age 21 years, 48% (472 of 977) at age 26 years, 46% (444 of 969) at age 32 years, 45% (429 of 955) at age 38 years, and 44% (407 of 927) at age 45 years.

**Figure 1.**
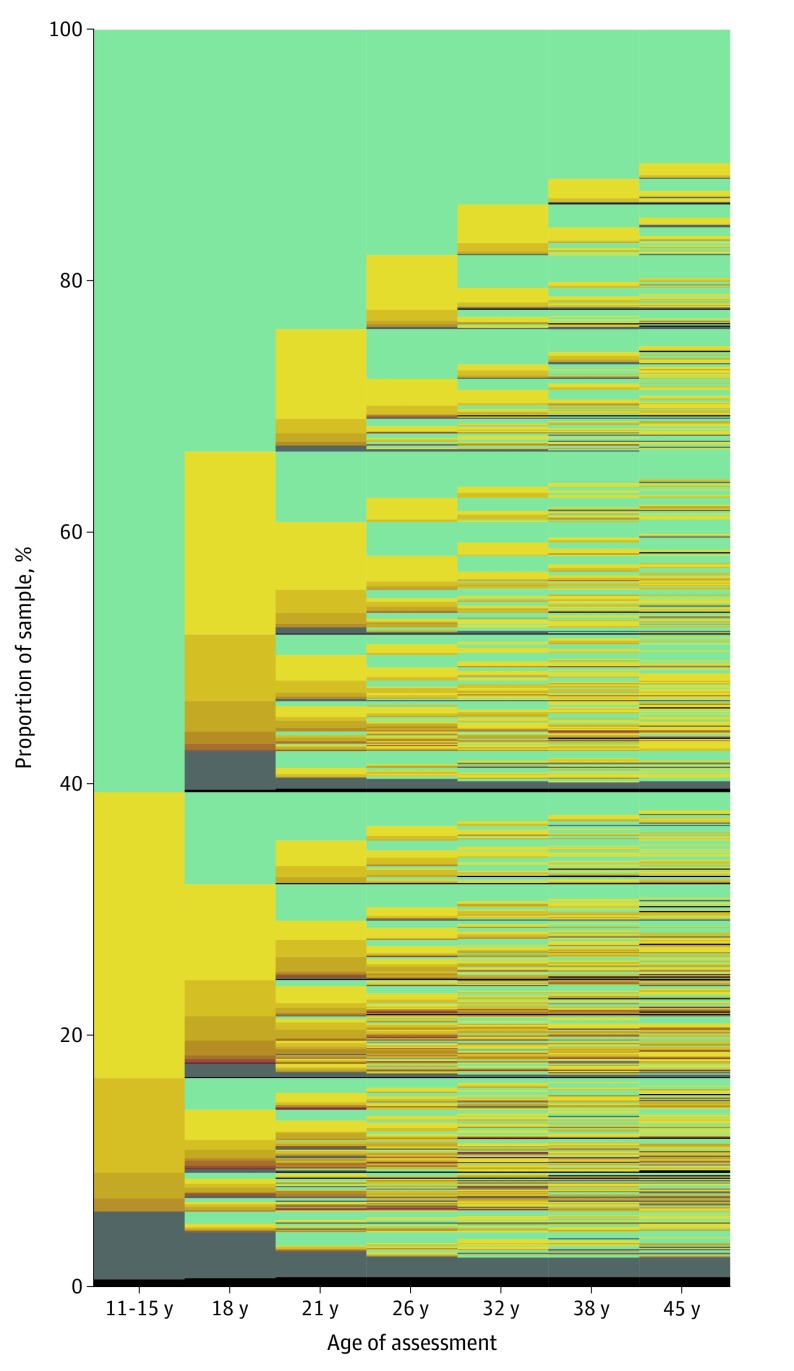
Natural History of Mental Disorders in a Cohort of 1037 Individuals The graph is made up of a thin line for each individual in the Dunedin Study stacked together to show the 1037 cohort members, followed from age 11 to age 45 years. Columns are assessment ages. Green signifies the absence of a mental disorder. Yellow signifies that an individual met the criteria for a psychiatric diagnosis at a given assessment age; as the yellow deepens into orange and brown, it signifies a greater number of concurrent disorders diagnosed for that individual. Gray signifies that a study member was missing at that assessment age. Black signifies that a study member had died.

**Figure 2.**
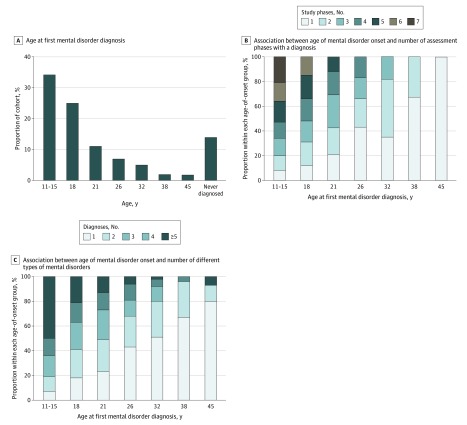
Early-Onset Mental Disorders and Their Sequelae A, Assessment age at which participants first met diagnostic criteria for a mental disorder. B, Proportion of participants within each onset age who met diagnostic criteria for a mental disorder in 1, 2, 3, 4, 5, 6, or 7 assessment windows. C, Proportion of participants within each onset age who met diagnostic criteria for 1, 2, 3, 4, or 5 or more different types of mental disorders in subsequent years, up to midlife.

Figure 1 visualizes the raw data for the 1037 original cohort members, followed from ages 11 to 45 years. The figure reveals 3 patterns. First, most participants first received a disorder diagnosis as a teenager (Figure 1). Approximately one-third (346 of 1013 [34%]) of the cohort experienced initial onset of a disorder by age 15 years, and nearly two-thirds (600 of 1013 [59%]) experienced initial onset of a disorder by age 18 years. Virtually no participants received a first diagnosis at age 45 years (Figure 2A). Second, early onset was associated with more years with a disorder and more comorbid disorders (Figure 1). Regarding duration, participants with early-onset disorders subsequently met diagnostic criteria at more past-year assessments (*r* = 0.71; 95% CI, 0.68-0.74; *P* < .001) (Figure 2B). Regarding comorbidity, participants with early-onset disorder subsequently met criteria for more diverse disorder types (*r* = 0.64; 95% CI, 0.60-0.67; *P* < .001) (Figure 2C). These associations remained after correction for the number of years available for observation between each participant’s first onset and study end (eAppendix 2 in the Supplement). Third, almost everyone eventually experienced a disorder (Figure 1). Cumulatively, by age 45 years, 86% (869 of 1013) of the cohort met the criteria for at least 1 disorder. This seemingly high lifetime prevalence is not unique to this cohort; it matches prevalence reports from multiple psychiatric-epidemiology studies around the world (eAppendix 5 in the Supplement).

**Figure 3.**
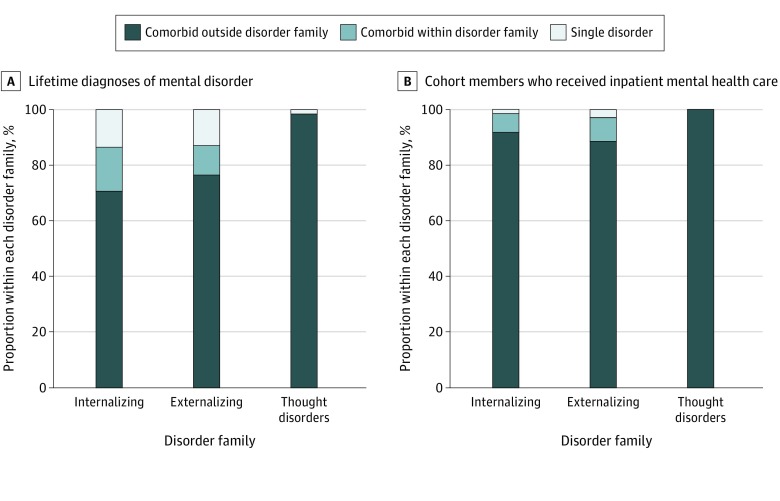
Lifetime Diagnoses of Single and Comorbid Disorders A, Information about the 869 participants who were ever diagnosed by the study with a mental disorder; 712 met criteria for an internalizing disorder, 625 met criteria for an externalizing disorder, and 177 met criteria for a thought disorder. Each bar is divided according to whether, over the course of their lifetime, participants also met criteria for another disorder outside that family of disorders, met criteria for another disorder within that family of disorders, or met criteria for just a single disorder. B, Same analysis restricted to the 83 cohort members who received inpatient mental-health services: 74 met criteria for an internalizing disorder, 70 met criteria for an externalizing disorder, and 41 of 83 met criteria for a thought disorder.

Participants characterized by only 1 pure disorder were atypical. For example, among participants ever diagnosed with an internalizing disorder (Figure 3A), most (503 of 712 [70%]) also experienced externalizing or thought disorders and another 16% (113 of 712) had multiple kinds of internalizing disorders. This left only 14% (96 of 712) of participants with internalizing disorders who experienced only 1 pure type of internalizing disorder, such as depression or 1 anxiety disorder type. Of interest, 75% (72 of 96) of these participants met the criteria for a disorder at only 1 assessment age. The same cross-family pattern was observed among participants ever diagnosed with an externalizing disorder (Figure 3A); most (478 of 625 [77%]) also experienced internalizing or thought disorders and another 11% (67 of 625) had multiple kinds of externalizing disorders. This left only 13% (80 of 625) of participants with externalizing disorders who experienced only 1 pure type of externalizing disorder, such as attention-deficit/hyperactivity disorder or cannabis dependence. Of interest, 71% (57 of 80) of these participants met the criteria for a disorder at only 1 assessment age. Fewer than 2% (3 of 177) of participants with a thought disorder experienced only 1 pure type of thought disorder, such as obsessive-compulsive disorder, mania, or schizophrenia (Figure 3A). To approximate hospital-register data, we restricted this analysis to 83 cohort members who had ever received inpatient treatment (Figure 3B); inpatients who had 1 exclusive diagnosis lifetime were rare (eAppendix 6 in the Supplement).

### The Ebb and Flow of Mental Disorders Over Decades

Cross-sectionally, internalizing, externalizing, and thought disorder families co-occurred at every assessment (eAppendix 7 in the Supplement). Sequentially, participants with a disorder in any of the 3 diagnostic families at 1 specific age were at higher risk for both other diagnostic families at subsequent ages, and all disorders were associated with an elevated risk for all other disorders (eAppendix 8 in the Supplement). Lifetime comorbidity thus accumulated from adolescence to age 45 years. At ages 11 to 15 years, 32% (110 of 346) of participants with a disorder had comorbid diagnoses, but by age 45 years, 85% (737 of 869) of participants with a disorder had accumulated comorbid diagnoses (eAppendix 9 in the Supplement).

**Figure 4.**
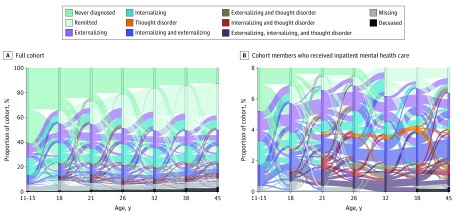
Ebb and Flow of Mental Disorders Sankey diagrams show cohort members’ shifting diagnoses from 1 assessment phase to the next, from ages 11 to 15 years to age 45 years. The colors of the horizontal bands divide the diagram into different psychiatric statuses, as indicated in the key. The heights of the horizontal bars show the prevalence of different statuses at each assessment phase. A, Information for the full cohort of 1037 participants. B, Analysis restricted to 83 participants who received inpatient mental-health services (8% of the cohort). Note that it is possible to follow groups across contiguous adjacent assessments, not across the entire panel.

Figure 4A depicts the movement of participants in and out of diagnoses. Four findings stand out. First, the number of participants surviving to midlife without a disorder diminished with time (also seen in Figure 1). Second, intermittent remission occurred, as shown by paths leading into and out of disorder-free periods. Third, there was some preservation of disorder across age. Fourth, there was substantial movement between diagnostic families in every direction at every age. Tracing all 1037 participants across time revealed 692 mental disorder life history patterns, of which 605 (87.4%) were unique to 1 person (Figure 4A). To approximate hospital-register data, we restricted the analysis to participants who received inpatient mental health services; movement between diagnostic families was even more pronounced among these 83 individuals (Figure 4B). Each participant with inpatient treatment followed a unique mental disorder life history pattern (eAppendix 10 in the Supplement).

### Mental Disorder Life Histories: Age at Onset, Duration, Diversity, and the p-Factor

Participants’ age at the onset of a disorder, duration in terms of number of assessment ages with a disorder, and diversity of diagnoses were positively intercorrelated (onset age with number of assessment ages, *r* = 0.71 [95% CI, 0.68-0.74]; onset age with comorbid variety, *r* = 0.64 [95% CI, 0.60-0.67]; number of assessment ages with comorbid variety, *r* = 0.83 [95% CI, 0.81-0.85]; all *P* < .001). We used confirmatory factor analysis of symptom-level data to summarize participants’ mental disorder life histories. A model that specified a general factor of psychopathology, the p-factor, fit the data set well, and symptom factor loadings were all positive and high, with a mean loading of 0.612 (range, 0.300-0.976; all *P *<* *.001) (eAppendix 4 in the Supplement). Participants with higher p-factor scores experienced younger age at onset (*r* = 0.48; 95% CI, 0.43-0.52), greater number of assessment ages with a disorder (*r* = 0.69; 95% CI, 0.66-0.72), and greater diversity of diagnoses (*r* = 0.76; 95% CI, 0.73-0.78) (eAppendix 4 in the Supplement).

### Mental Disorder Life Histories and Health of the Brain

**Figure 5.**
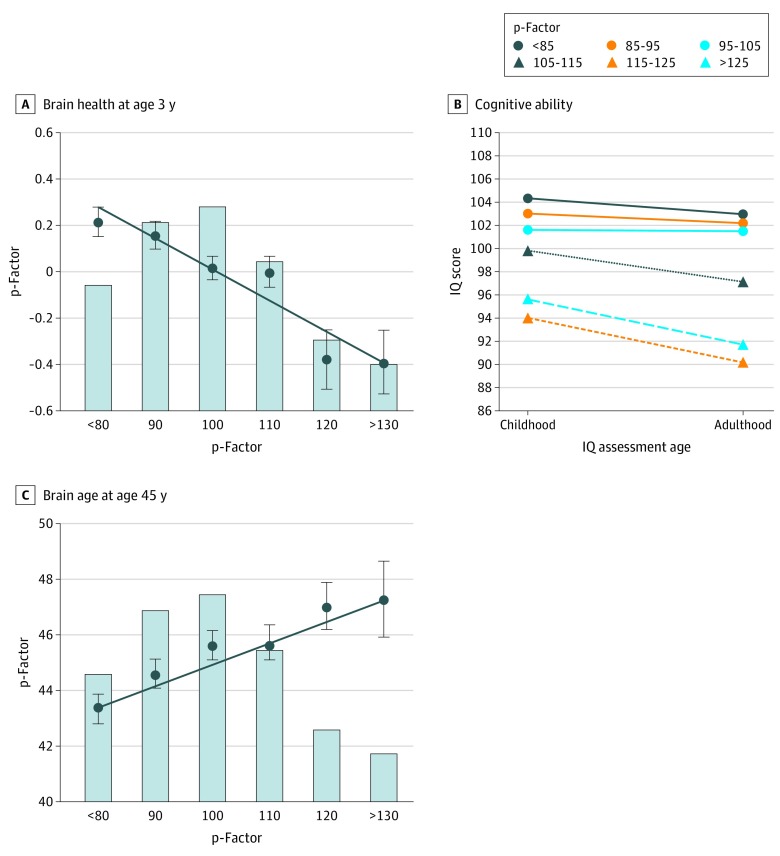
Origins and Sequelae of the p-Factor Graphs show that compromised brain health at age 3 years was associated with higher p-factor scores (A) and that higher p-factor scores were associated with more decline in cognitive ability from childhood to adulthood (B) and older brain age by midlife (C). In each panel, the p-factor score is standardized to a mean (SD) of 100 (15), and higher p-factor scores indicate more generalized psychopathology. In A and C, the bars of the histograms graph the proportions of the sample at different levels of the p-factor score (midpoint of 10-point bands): less than 85 (163 participants [16.3%]), 85 to 95 (237 participants [23.7%]), 95 to 105 (259 participants [25.9%]), 105 to 115 (189 participants [18.9%]), 115 to 125 (91 participants [9.1%]), and greater than 125 (61 participants [6.1%]). The circles and SE bars show the mean scores of individuals in each p-factor score group; these groups have been clumped solely for graphing purposes (with group size >50). The regression lines in A and C show the association between the p-factor score and its childhood correlates and adult sequelae. The regression coefficients reported in the text are based on the full distribution of p-factor scores (see eAppendix 11 in the Supplement for scatterplots).

Children who grew up to score higher on the p-factor performed more poorly on neurocognitive examinations at age 3 (*r* = −0.18; 95% CI, −0.24 to −0.12; *P* < .001) (Figure 5A). Later in childhood, they had lower Wechsler Intelligence Scale for Children–Revised IQ scores (*r* = −0.19; 95% CI, −0.25 to −0.13; *P* < .001). Their cognitive functions continued to decline, as revealed by lower Wechsler Adult Intelligence Scale–IV IQ at age 45 years compared with their childhood IQ (*r* = −0.11; 95% CI, −0.17 to −0.04; *P* < .001) (Figure 5B). By age 45 years, participants with higher p-factor scores showed older brain age (*r* = 0.14; 95% CI, 0.07 to 0.20; *P* < .001) (Figure 5C). Figure 5 shows that compared with cohort peers with the lowest p-factor scores, participants with the highest p-factor scores had brain health 0.61 SD weaker, child-to-adult cognitive decline 3.8 IQ points steeper, and midlife brain-age 3.9 years older (eAppendix 11 and eAppendix 12 in the Supplement).

## Discussion

Participants in this 4-decade study of a population-representative cohort had mental disorder life histories that could not be adequately characterized by a diagnosis at 1 point in time. This research advances knowledge in 5 ways. First, this study confirmed prior reports^40,41^ that most individuals who experience mental disorder have first onset as juveniles (34% before age 15 years; 59% before age 18 years). Second, it further confirmed the high lifetime prevalence reported by multiple longitudinal cohort studies that use repeated psychiatric assessments to counteract undercounting caused by retrospective recall failure; a previous review^42^ concluded that most of the population eventually experiences mental disorder, whereas people who sustain enduring mental health are rare exceptions (14% in our cohort). Third, we replicated Danish-register findings that patients in psychiatric clinics tend to experience diverse disorders in turn, and every disorder is associated with elevated risk for every other disorder.^20^ We expanded on that prior work by providing initial evidence that outside-family comorbidity is characteristic of the general population, as well as registered patients. In contrast to assumptions of diagnosis-specific research and clinical protocols, we found evidence that virtually no one gets and keeps 1 pure diagnosis type. Fourth, this study applied a novel life-course approach to longitudinal data about mental disorders. Three key life-course parameters tended to converge in the same individuals: younger age at disorder onset, more years’ duration of disorder, and more diverse types of comorbid disorders (even after controlling for each participant’s years after onset). A single dimension derived from all symptoms reported over multiple decades, the p-factor, summarized the differences between individuals in their mental disorder life history. Fifth, these life histories were antedated by compromised brain health in early childhood (whether genetically inherited or acquired from adverse experiences), were accompanied by cognitive decline from childhood to midlife, and were associated with older brain age measured via structural neuroimaging at midlife.

### Limitations and Strengths

This study has limitations. First, the findings come from a predominantly white sample, 1 country, and 1 historical period. However, previous mental health findings from this cohort have been replicated in other countries, including evidence about lifetime prevalence and the structure of the p-factor.^24,43^ Moreover, this analysis replicated Danish-register findings.^20^ Second, our analysis was left-hand censored at age 11 years and right-hand censored at age 45 years. Third, Dunedin participants have lived through the changes from the *DSM-III *to the *DSM-5*; some disorders’ criteria have changed, and interview questions were accordingly updated. As such, the findings reflect the changing health care practices during participants’ lives. Fourth, and relatedly, the study did not assess disorders that at the time were assumed to have very low base rates (eg, childhood autism). Fifth, many analyses treated disorders as discrete categories, despite awareness that diagnostic thresholds are decision-making conventions. However, our analyses summarizing mental disorder life histories with the p-factor used symptom-level data, exploiting meaningful information above and below diagnostic thresholds. Sixth, although unreliability may influence diagnostic decisions both in research and in clinical practice, the Dunedin study’s diagnostic reliability is sufficient for research, and unreliability is not the reason we observe shifting among different successive disorders across the life course (eAppendix 8 in the Supplement). Moreover, the same findings emerge from Dunedin mental disorder life histories as from Danish registered discharge diagnoses.^20^

This study has implications for public understanding. Mental disorder eventually affects almost everyone. Some mental disorder life histories resemble a fractured leg or influenza, disabling but short-lived. Other mental disorder life histories become chronic or recurrent. However, people meeting diagnostic criteria experience impaired functioning and many absorb health care resources. Public health education about the ubiquity of disorder could reduce stigma and promote earlier and increased treatment uptake, facilitating prevention. Rather than viewing mental disorders as rare, members of the general public should expect at least 1 bout of mental disorder in their lifetime.

There are implications for prevention. Juvenile onset was highly prevalent and portended more years of disorder, greater diversity of comorbid disorders, and reduced likelihood of recovery, which were linked to cognitive decline and older structural brain age by midlife. These findings advise directing more mental health resources toward pediatric efforts to prevent mental disorder, especially because only a minority of children with disorder receive effective treatment. Ubiquitous juvenile onset also means that newly presenting adult patients almost certainly experienced prior disorder (even if their memory fails them), and those disorders may have looked quite different from the current disorder. Of course, clinicians will not have the benefit of their patients being enrolled in a 4-decade longitudinal study. An obvious caveat is that clinicians must treat the disorder that appears before them, offering relief for the patient’s current complaint. The life-course approach thus has 2 clinical implications. First, looking to the past, it places priority on expert history taking to support strategic treatment planning.^44,45^ Second, looking to the future, because many patients will go on to experience diverse disorders, therapy cannot just mitigate the presenting symptoms, but must also build skills for maintaining enduring mental health. The life-course approach makes transdiagnostic interventions high priority.

There are implications for etiological research. First, finding specific causes matched to specific disorders has been a highly desirable but elusive research goal,^46^ but the present findings suggest that causal specificity may be unrealizable because mental disorder life histories include diverse disorders. The life history approach explains why genetic,^9,10,11^ neuroscience,^4,6,7^ and risk-factor research^12,13^ point to shared causes underlying an array of disorders.^47^ Second, our findings suggest that research can be misled by cross-sectional designs. Particularly problematic are case-control studies that enroll patients on the basis of the current disorder in their mental disorder life history (unaware of other past and future disorders) and compare them against currently well controls (who may have been unwell in the past and may become unwell in the future).^48^ A third implication is that etiological research might productively embrace dimensions that quantify variation in mental disorder life histories. The findings here suggest that dimensions such as age at onset, duration, diversity—or the p-factor—may reflect patients’ lives (especially in inpatient settings) better than any particular differential diagnosis can.

## Conclusions

Much research shows that sustained mental wellness is rare, and this study shows that presentation with only 1 diagnosis—and even 1 diagnostic family—is rarer still, suggesting that it may be time to adopt a life-course perspective on mental disorders. The life-course framework orients research away from the cause of a single disorder at 1 point in time toward studying the dynamics of mental disorder life histories. We hope that the findings reported here encourage research to design tools to assess an individual’s life-course vulnerability to psychopathology, identify causes of this vulnerability, explain why this vulnerability manifests in different diagnoses at different points in the life course, and develop transdiagnostic preventions.

## Notes

Supplement. **eAppendix 1.** Sample **eAppendix 2**. Assessing Psychopathology **eAppendix 3**. Measuring Brain Function Across the Life Course: Age-3 Brain Health, Child and Adult Cognitive Functioning, Child-to-Adult Cognitive Decline, and Accelerated Brain Aging **eAppendix 4**. Modeling the Structure of Psychopathology **eAppendix 5**. Prevalence of Mental Disorder in the Dunedin Study **eAppendix 6**. Does Anyone Have Just One Exclusive Diagnosis? **eAppendix 7**. Cross-Sectional Comorbidity **eAppendix 8**. Sequential Comorbidity **eAppendix 9. **Lifetime Comorbidity **eAppendix 10. **The Ebb and Flow of Mental Disorders Among Participants Who Received Inpatient Mental-Health Services **eAppendix 11.** Correlations Between Measures of Mental-Disorder Life-Histories and Measures of Brain Function From Childhood to Midlife **eAppendix 12. **MPlus Syntax **eReferences.**
