# Supplementary material for: Longitudinal Assessment of Mental Health Disorders and Comorbidities Across 4 Decades Among Participants in the Dunedin Birth Cohort Study
Source: JAMA Netw Open. 2020 Apr 21;3(4):e203221. doi: 10.1001/jamanetworkopen.2020.3221 (PMC7175086; doi:10.1001/jamanetworkopen.2020.3221)
Supplement: Supplement. — eAppendix 1. Sample eAppendix 2. Assessing Psychopathology eAppendix 3. Measuring Brain Function Across the Life Course: Age-3 Brain Health, Child and Adult Cognitive Functioning, Child-to-Adult Cognitive Decline, and Accelerated Brain Aging eAppendix 4. Modeling the Structure of Psychopathology eAppendix 5. Prevalence of Mental Disorder in the Dunedin Study eAppendix 6. Does Anyone Have Just One Exclusive Diagnosis? eAppendix 7. Cross-Sectional Comorbidity eAppendix 8. Sequential Comorbidity eAppendix 9. Lifetime Comorbidity eAppendix 10. The Ebb and Flow of Mental Disorders Among Participants Who Received Inpatient Mental-Health Services eAppendix 11. Correlations Between Measures of Mental-Disorder Life-Histories and Measures of Brain Function From Childhood to Midlife eAppendix 12. MPlus Syntax eReferences. [file jamanetwopen-3-e203221-s001.pdf]

Supplementary Online Content

Caspi A, Houts RM, Ambler A, et al. Longitudinal assessment of mental health disorders and comorbidities across 4 decades among participants in the Dunedin birth cohort study. *JAMA Netw Open*. 2020;3(4):e203221. doi:10.1001/jamanetworkopen.2020.3221

eAppendix 1. Sample
eAppendix 2. Assessing Psychopathology
eAppendix 3. Measuring Brain Function Across the Life Course: Age-3 Brain Health, Child and Adult Cognitive Functioning, Child-to-Adult Cognitive Decline, and Accelerated Brain Aging
eAppendix 4. Modeling the Structure of Psychopathology
eAppendix 5. Prevalence of Mental Disorder in the Dunedin Study
eAppendix 6. Does Anyone Have Just One Exclusive Diagnosis?
eAppendix 7. Cross-Sectional Comorbidity
eAppendix 8. Sequential Comorbidity
eAppendix 9. Lifetime Comorbidity
eAppendix 10. The Ebb and Flow of Mental Disorders Among Participants Who Received Inpatient Mental-Health Services
eAppendix 11. Correlations Between Measures of Mental-Disorder Life-Histories and Measures of Brain Function From Childhood to Midlife
eAppendix 12. MPlus Syntax
eReferences

This supplementary material has been provided by the authors to give readers additional information about their work.

© 2020 Caspi A et al. *JAMA Network Open.*

eAppendix 1. Sample

Participants are members of the Dunedin Study, a longitudinal investigation of health and behavior in a complete birth cohort. The 1,037 (535[52%] male) participants were all individuals born between April 1972-March 1973 in Dunedin, New Zealand (NZ), who participated in the first assessment at age 3 years, representing 91% of participants who were eligible based on residence in the province [1]. The cohort represented the full range of socioeconomic status on NZ's South Island and in adulthood matches the NZ National Health and Nutrition Survey on key health indicators (e.g., BMI, smoking, GP visits) and matches the NZ Census of citizens the same age on educational attainment [2]. The cohort is primarily white (93%), matching South Island demographics. Assessments were carried out at birth and ages 3, 5, 7, 9, 11, 13, 15, 18, 21, 26, 32, 38, and most recently, 45 years, when 94% of the 997 Study members still alive took part. At each assessment, each Study member is brought to the research unit for a full day of interviews and examinations. Written informed consent was obtained from cohort participants, and study protocols were approved by the institutional ethical review boards of the participating universities.

Beginning at age 11 years, Study members have been interviewed privately by health professionals about their mental health and psychiatric diagnoses have been made according to the Diagnostic and Statistical Manual of Mental Disorders (DSM). Pediatric neurocognitive examinations were carried out at age 3, neuropsychological testing was carried out in both childhood and adulthood, and neuroimaging was performed at age 45 when brain age was estimated.

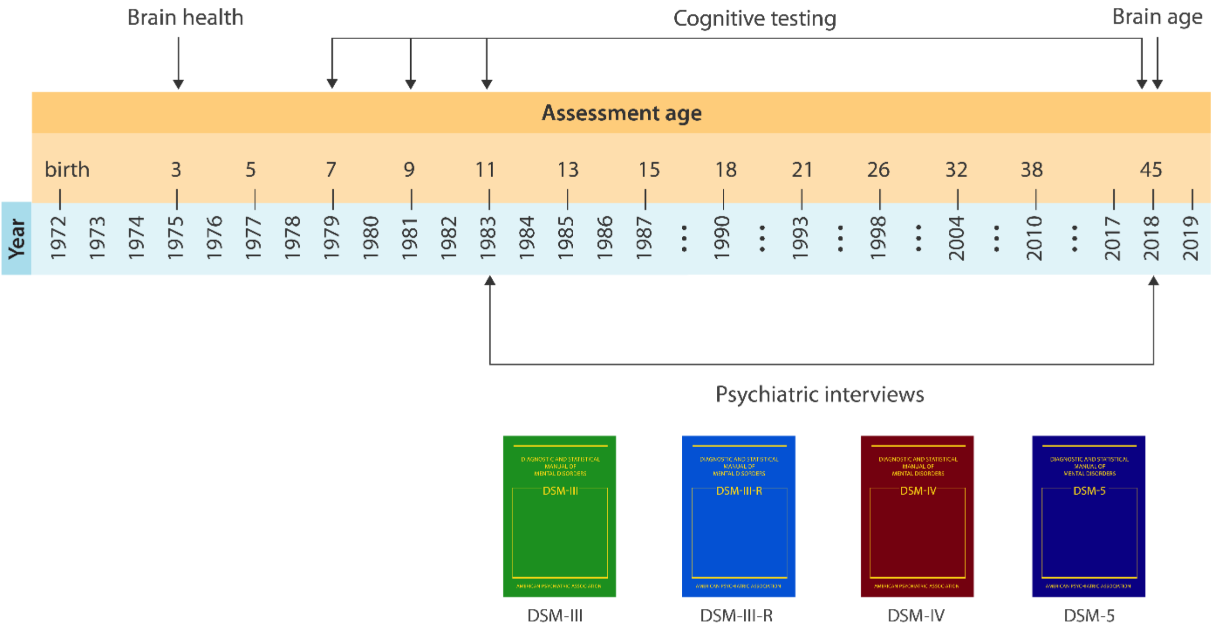

© 2020 Caspi A et al. *JAMA Network Open.*

eAppendix 2. Assessing Psychopathology

Mental disorders are disturbances in thought, behavior, and emotion that interfere with or limit social, family, educational, or work activities. In the Dunedin Study, these were identified according to the criteria of the Diagnostic and Statistical Manual of Mental Disorders (DSM). The Dunedin Study longitudinally ascertains mental disorders using a strategy akin to experience sampling: At every assessment phase, Study members were interviewed about past-year symptoms. Past-year reports maximize recall because recollections over longer periods are less accurate. It is possible that past-year reports separated by 1 to 6 years miss episodes of mental disorder occurring only in gaps between assessments. We tested for this possibility by using life-history calendar interviews at each assessment [3] to ascertain indicators of mental disorder occurring in the gaps between assessments, including inpatient treatment, outpatient treatment, or spells taking prescribed psychiatric medication (indicators that are salient and recalled more reliably than individual symptoms). Life-history calendar data indicated that virtually all Study members having a disorder consequential enough to be associated with treatment have been detected in our net of past-year diagnoses. Specifically, we identified only 17 people who reported treatment but had not been captured in our net of diagnoses. Of the missed cases, 5 reported short-term treatment for post-partum depression, 1 reported treatment for seasonal affective disorder, 1 died following a suicide attempt, and 10 reported they were treated by a family doctor for anxiety or depression.

Psychiatric interviews were carried out by health professionals (psychiatric nurses, psychiatric social workers, clinical psychologists, GPs, and psychiatrists, all of whom had professional clinical experience), not lay interviewers. Interviewers were kept blind to cohort members' prior mental health data. At ages 11, 13, and 15, interviews were carried out with the Diagnostic Interview Schedule for Children-Child Version [(3)]. These disorders were assessed in childhood: Externalizing (ADHD, Conduct Disorder) and Internalizing (Depression, Anxiety and Fears [including Separation Anxiety, Overanxiety, Social Phobia, Simple Phobia]). At ages 18, 21, 26, 32, 38, and 45, interviews were carried out with the Diagnostic Interview Schedule [4,5]. These disorders were assessed in adulthood: Externalizing (ADHD, Conduct Disorder, Alcohol Dependence, Tobacco Dependence, Cannabis Dependence, Other Drug Dependence), Internalizing (Generalized Anxiety Disorder, Depression, Fears [including Social Phobia, Simple Phobia, Agoraphobia, Panic Disorder], Eating Disorders [including Bulimia and Anorexia], PTSD), and Thought disorders (Obsessive-Compulsive Disorder, Mania, Schizophrenia). To allow the study of comorbidity, multiple diagnoses could be assigned to a participant at once. However, DSM exclusionary criteria were applied (e.g., hallucinations better explained by drug use were not counted toward schizophrenia; generalized anxiety disorder was not diagnosed if the anxiety stemmed solely from fear about public speaking).

The diagnoses were made using computerized algorithms matching the DSM criteria, and additionally requiring self-reported impairment ratings. In the younger years, parent and teacher data were brought in to confirm presence of key symptoms and impairments. (This followed best clinical practice for juveniles.) In the later years, for disorders where self-reports can be compromised by lack of insight (such as schizophrenia, mania), we also turned to information from additional sources, such as interviews with parents, systematic questionnaires mailed to informants who know the study member well (present data for 97% of the cohort), standardized clinical staff ratings (of observed behavior, such as poor grooming or bizarre speech, during the day of assessment), and medical records for each cohort member from the New Zealand national health system. In the case of schizophrenia and mania, narrative dossiers of symptoms were reviewed by two experienced psychiatrists to achieve diagnostic consensus. These details are reported in our previous publications (see, e.g.[6]).

The chart on the next page shows the age at which each disorder was assessed. Although each disorder was not assessed at every age, each disorder was assessed on at least three occasions.

© 2020 Caspi A et al. *JAMA Network Open.*

	Age diagnosis made						
	11-15y	18y	21y	26y	32y	38y	45y
**EXTERNALIZING DISORDERS**							
ADHD	●	●				●	●
Conduct Disorder	●	●	●	●	●	●	●
Alcohol Dependence		●	●	●	●	●	●
Tobacco Dependence		●	●	●	●	●	●
Cannabis Dependence		●	●	●	●	●	●
Drug Dependence				●	●	●	●
**INTERNALIZING DISORDERS**							
Anxiety (GAD)	●	●	●	●	●	●	●
Depression (MDE)	●	●	●	●	●	●	●
Fears (any Panic, SIP, SOP, Agoraphobia)	●	●	●	●	●	●	●
Eating Disorder (any bulimia, anorexia)		●	●	●			
PTSD				●	●	●	●
**THOUGHT DISORDERS**							
Obsessive Compulsive Disorder		●	●	●	●	●	●
Mania			●	●	●	●	●
Schizophrenia			●	●	●	●	●

Up to age 15, diagnoses were made according to DSM-III [7]; at ages 18 and 21, according to DSM-III-R [8]; at ages 26, 32, and 38, according to the DSM-IV [9]; at age 45 according to the now-current DSM-V [10] (with the exception of substance-dependence disorders which were diagnosed according to DSM-IV, given that DSM-V dropped the distinction between abuse and dependence). This is a limiting factor of our research because diagnostic criteria for some, but not all, disorders have changed a bit over the course of the past 35 years. It is also reality; the length of the Dunedin Study means that Study members have lived through multiple versions of psychiatric nosologies. We do not have the ability to always match past interviews to current nosologies or current interviews to past nosologies. As such, our report about the natural history of mental health reflects the lived experiences of Study members.

To describe the longitudinal patterns of mental disorder we focused on three developmental parameters: age-of-onset, duration (number of phases during which diagnostic criteria were met), and diversity (number of disorder types whose criteria were met). Figure 2 in the Main Article shows that these three key developmental parameters of mental-disorder life-histories were inter-correlated: age-of-onset was correlated with the number of assessment phases during which diagnostic criteria were met ($r=.71$ [95%CI:.68,.74], $p<.001$), with meeting criteria for more different types of disorders ($r=.64$ [95%CI:.60,.67], $p<.001$), and number of assessment phases during which diagnostic criteria were met was correlated with meeting criteria for more different types of disorders ($r=.83$ [95%CI:.81,.85], $p<.001$). The Table on the next page shows these same data in a tabular form. Column 1 shows the assessment age at which participants first met diagnostic criteria for a mental health disorder. Columns 2 and 3 show the sequelae of early onset. Early onset was associated with a greater likelihood of meeting diagnostic criteria at more subsequent 12-month assessment windows, up to midlife (column 2) and with meeting criteria for more different types of psychiatric disorders in subsequent years, up to midlife (column 3).

© 2020 Caspi A et al. *JAMA Network Open.*

	Age of first mental health diagnosis		Number of study phases with mental health diagnosis		Number of mental health diagnoses	
Assessment Age	N	% (95% CI)	M (SD)	Range	Mean (SD)	Range
11-15 y	346	34.2% (31.2%, 37.1%)	4.48 (1.94)	1 - 7	4.77 (2.48)	1 - 12
18 y	254	25.1% (22.4%, 27.7%)	3.61 (1.62)	1 - 6	3.20 (1.84)	1 - 10
21 y	111	11.0% (9.0%, 12.9%)	2.79 (1.29)	1 - 5	2.77 (1.63)	1 - 10
26 y	70	6.9% (5.3%, 8.5%)	2.09 (1.14)	1 - 4	2.14 (1.30)	1 - 6
32 y	49	4.8% (3.5%, 6.2%)	1.84 (0.72)	1 - 3	1.80 (1.02)	1 - 5
38 y	24	2.4% (1.4%, 3.3%)	1.33 (0.48)	1 - 2	1.38 (0.58)	1 - 3
45 y	15	1.5% (0.7%, 2.2%)	1.00 (0.00)	1	1.40 (1.06)	1 - 5
Never dx	144	14.2% (12.1%, 16.4%)	--	--	--	--

Correction for observation window. It is possible that diversity of comorbid diagnoses could be a function of age of-onset, if individuals with older age-of-onset had fewer remaining waves of the study for diagnoses to be made. To correct for this, we calculated each individual's personal rate of diagnoses, by dividing their number of diagnoses by the 'n' of years between their onset age and the end of the study. This rate is referred to in the economics literature as a personal lambda. Next we re-estimated the association between age-of-onset and lifetime 'n' of total diagnoses. To perform this analysis, we had to omit those Study members who never met diagnostic criteria for a mental disorder and those who first met diagnostic criteria for a mental disorder at age 45 years. In the remaining subset of Study members (who had onset between 11 and 38 years), the association between age-of-onset and future n of disorders was r=.22 (95%CI: .17, .28), p<.001.

Additional details about mental disorder diagnoses in the Dunedin cohort. A reviewer inquired about the rates of schizophrenia and OCD in the Dunedin cohort.

The lifetime rate of schizophrenia in the Dunedin cohort is 3.7%. We have published our method of diagnosing schizophrenia in multiple publications over the past 2 decades [6,11]. It is believed that the prevalence of schizophrenia should be 1%, but, as we have discussed previously, there is a wide confidence interval around this 1% estimate. The Dunedin cohort's prevalence rate should be understood in the context of four methodological aspects of our study. First, our birth cohort, with its low attrition rate, allows us to count individuals with schizophrenia disorders overlooked by previous surveys. Individuals with psychotic disorders often decline to participate in surveys or die prematurely, and in addition surveys often exclude homeless or institutionalized individuals with psychosis. Our study assesses all of these groups missing from other surveys. Second, our cohort members are all from one city in the South Island of New Zealand. It is possible, given the known geographical variation in rates of schizophrenia, that the prevalence is somewhat elevated there. No comparable data exist to compare prevalence rates of schizophrenia in New Zealand to rates in other countries, but New Zealand has the highest prevalence of suicide worldwide and this fact could be consistent with a locally elevated prevalence of severe mental health conditions. Third, estimates of schizophrenia tend to be based on patients in clinical registers, but registers omit many community-dwellers whose disorder goes untreated. We note that over half of those diagnosed by the Dunedin Study were confirmed by receipt of treatment. By age 45, 2% of the cohort (N=20) met full DSM criteria for schizophrenia and had also been hospitalized for schizophrenia, according to our official New Zealand health system administrative record searches. However, an additional 1.7% (N=17) met all DSM criteria for schizophrenia, had auditory hallucinations by self-report (a criterion more strict than DSM), and suffered significant life impairment according to their informants. These 17 individuals had not, to our knowledge, been treated yet specifically for psychotic illness (those 20 treated and 17 not treated do not differ on cognitive status or symptom picture). Fourth, our research diagnoses did not make fine-grained distinctions among subtypes of psychotic disorders (e.g., schizophrenia versus schizoaffective psychotic disorder). Thus, the cohort members diagnosed with schizophrenia here might not be considered by all clinicians to have exclusively pure schizophrenia, which is what the oft-cited 1% lifetime prevalence rate is intended to reflect. Dunedin diagnoses of psychosis have been confirmed by consensus review by 2 psychiatrists.

© 2020 Caspi A et al. *JAMA Network Open.*

The lifetime rate of OCD in the Dunedin cohort is 15%. It is believed that the prevalence rate should be around 2-3%. This belief is probably based on the NCS-R estimate. The NCS-R estimate is based on lifetime retrospective reports, which are known to undercount. It has been shown in multiple longitudinal cohort studies that lifetime prevalence rates in retrospective surveys are undercounted by at least half for many conditions; in fact, this literature is presented for readers in eAppendix 5 in our Supplement. How this discrepancy in lifetime prevalence between (a) one-off retrospective surveys and (b) cumulative prospective longitudinal studies happens can be easily seen in the Dunedin Study. The 12-month rates of OCD are presented in the table below, in gray. They range from 2% (in midlife adults) to 7% (in young people). OCD disorders are fairly stable in the cohort. That is, people who are diagnosed with OCD at one age are statistically more likely to be diagnosed with OCD at subsequent ages. This can be seen in the table below, by the transition matrix of odds ratios (transition ORs) which follows the simplex-like pattern one expects to see in longitudinal data. But there is also change over time (many people remit from OCD and new incident cases of OCD also accumulate). The result is that through multiple assessments, we end up with a total number of 150 who met diagnostic criteria for OCD at least once during several decades. Indeed, this is one important point of our report. When one takes a longitudinal life-course perspective on mental disorders rather than a cross-sectional snapshot, we see that lifetime mental disorders are much more prevalent than previously assumed. This is an important public health message (which we have discussed in the past specifically in reference to OCD[12]).

Prevalence rates of OCD and transition odds ratios for OCD (ORs):

		Transition OR's				
	Prevalence	Age 18	Age 21	Age 26	Age 32	Age 38
Age 18	4.6%					
Age 21	6.1%	9.44				
Age 26	2.4%	6.78	11.50			
Age 32	1.8%	2.98	5.07	16.07		
Age 38	2.8%	1.92	1.85	6.44	25.82	
Age 45	3.2%	1.81	1.95	8.84	13.59	26.97

© 2020 Caspi A et al. *JAMA Network Open.*

eAppendix 3. Measuring Brain Function Across the Life Course: Age-3 Brain Health, Child and Adult Cognitive Functioning, Child-to-Adult Cognitive Decline, and Accelerated Brain Aging

Measuring age-3 brain health. At age 3 years, each child in the cohort participated in a 45-minute examination that included assessments of neurological soft signs, intelligence, receptive language, and motor skills, and afterwards the examiners (having no prior knowledge of the child) rated each child's behavior (all described in the Table below). Using this information, we created a summary factor score via confirmatory factor analysis which we termed brain health, a global index of the child's early neurocognitive status [13]. The model fit the data well, χ^2(N=1035, df=5) = 6.459, p = .2641, CFI = .999, TLI = .997, RMSEA = .017. Factor scores were output and standardized to a Mean = 0 and SD= 1.

Measure/Test	Description
Neurologic soft signs	At age three years, each child was examined by a pediatric neurologist for neurologic signs, including assessment of motility, passive movements, reflexes, facial musculature, strabismus, nystagmus, foot posture, and gait, based on procedures described by Touwen & Prechtl [14].
Peabody Picture Vocabulary Test	Intelligence was assessed at age three with the Peabody Picture Vocabulary test [15].
Receptive Language	Receptive language was assessed at age three using the Reynell Developmental Language Scales [(25)].
Motor Development	Motor development was assessed at age three years with the Bailey Motor Scales [16].
Lack of Control	Following the testing, each examiner rated the child's lack of control in the testing session, yielding a behavioral style factor, labeled Lack of Control [17], which characterized children who at age three years were labile, had low frustration tolerance, lacked reserve, were resistant, restless, impulsive, required attention, and lacked persistence in reaching goals.

Measuring cognitive functioning and cognitive decline. The Wechsler Intelligence Scale for Children – Revised (WISC-R) [18] was individually administered at ages 7, 9, and 11 years. IQ scores for the three ages were averaged [19].

The Wechsler Adult Intelligence Scale–IV (WAIS-IV) [20] was individually administered at age 45 years.

We measured cognitive decline by studying IQ scores at midlife after controlling for IQ scores in childhood. (As a sensitivity analysis, in addition to analyzing residualized change we also analyzed difference (change) scores, and obtained the same substantive and statistically-significant results.) We focus on change in the overall IQ given evidence that age-related slopes are correlated across all cognitive functions, suggesting that research on cognitive decline may be best focused on a highly reliable summary index, rather than focused on individual functions [21].

Measuring accelerated structural brain aging. At age 45 years, brain images were acquired from Study members using a Siemens Skyra 3T equipped with a 64-channel head/neck coil. We estimated Brain Age with a publicly available algorithm [22] which uses information about cortical anatomy and whole-brain functional connectivity to estimate the age of a person's brain relative to their chronological age. The algorithm has been shown to predict chronological age in multiple independent samples, although it has a documented tendency to underestimate chronological age by approximately 3 years among adults between chronological ages 44-46 (and for this reason we standardized the scores to the mean chronological age of the Dunedin Study members at the time of their scanning in the Phase-45 assessment) [23]. Deviations of predicted brain age upwards of chronological age are presumed to reflect accelerated brain aging.

© 2020 Caspi A et al. *JAMA Network Open.*

eAppendix 4. Modeling the Structure of Psychopathology

We have previously described the structure of psychopathology up to age 38 years [13]; here we extend these models to include the age 45 data.

We used symptom data from the 6 adult assessments, carried out at ages 18, 21, 26, 32, 38, and 45 years. We studied DSM-defined symptoms of the following disorders that were repeatedly assessed in our longitudinal study: ADHD, Conduct Disorder, Alcohol Dependence, Cannabis Dependence, Dependence on Hard Drugs, Tobacco Dependence (assessed with the Fagerström Test for Nicotine Dependence [24]), Depression, Generalized Anxiety Disorder, Fears/Phobias (Social Phobia, Simple Phobia, Agoraphobia, Panic Disorder), PTSD, Eating Disorders (Anorexia, Bulimia), Obsessive-Compulsive Disorder, Mania, and positive and negative Schizophrenia symptoms. Ordinal measures represented the number of the observed DSM-defined symptoms associated with each disorder. Fears/phobias were assessed as the count of diagnoses for simple phobia, social phobia, agoraphobia, and panic disorder that a study member reported at each assessment. Of the 14 disorders, 6 were not assessed at every occasion, but each disorder was measured at least three times (see eAppendix 2). Of the original 1,037 study members, we included 1,000 study members who had symptom count assessments for at least one age (845 study members had present symptom counts for all six assessments, 90 for five, 30 for four, 13 for three, and 14 for two). The 37 excluded study members comprised those who died (N=13) or left the Study (N=21) before age 18 or who had such severe developmental disabilities (N=3) that they could not be interviewed with the Diagnostic Interview Schedule.

Using Confirmatory Factor Analysis (CFA), we tested two standard models that are frequently used to examine the structure of psychopathology [25]: (a) a correlated-factors model and (b) a hierarchical or bifactor model. Data analysis syntax appears in the last section of this supplement. In CFA, latent continuous factors are hypothesized to account for the pattern of covariance among observed variables. Our CFAs were run as multitrait-multimethod models. In these models, observed variables represented each of the disorders with a symptom scale at each assessment age (e.g., alcohol dependence was measured with a symptom scale at ages 18, 21, 26, 32, 38, and 45). Each model also included method/state factors designed to pull out assessment-related variance (e.g., assessment-specific interviewer effects, assessment-specific study member mood effects) that was uncorrelated with the psychopathology factors of interest. Because symptom-level data are ordinal and have highly skewed distributions, we used polychoric correlations when testing our models. Polychoric correlations provide estimates of the Pearson correlation by mapping thresholds to underlying normally distributed continuous latent variables that are assumed to give rise to the observed ordinal variables. All CFA analyses were performed in MPlus version 8.3 [26] using the weighted least squares means and variance adjusted (WLSMV) algorithm.[27] We assessed how well each model fit the data using the chi-square value, the comparative fit index (CFI), the Tucker-Lewis index (TLI), and the root-mean-square error of approximation (RMSEA). CFI values greater than .95 and TLI values greater than 0.95 indicate good fit; RMSEA scores less than .05 are considered good [28].

The correlated-factors model (see Model A on next page) tests the hypothesis that there are latent trait factors, each of which influences a subset of the diagnostic symptoms. We tested three factors representing Externalizing (with loadings from ADHD, conduct disorder, alcohol, cannabis, tobacco, and other drug dependence), Internalizing (with loadings from MDE, GAD, fears/phobias, PTSD, and eating disorders), and Thought Disorder (with loadings from OCD, mania, and schizophrenia). The model fit the data well: χ^2(2465, N=1,000) = 4082.230, CFI = .933, TLI = .929, RMSEA = .026, 90% confidence interval (CI) = [.024, .027]. As shown in the Table on page 10, loadings on the three specific factors were all positive, generally high (all ps < .001), and averaged .790 – Externalizing: average loading = .743; Internalizing: average loading = .814; Thought Disorder: average loading = .844. Correlations between the three factors were all positive and ranged from .420 between Internalizing and Externalizing to .847 between Internalizing and Thought Disorder. Thus, this model confirmed that three correlated factors (i.e., Internalizing, Externalizing, and Thought Disorder) explain well the structure of the disorder symptoms examined across 27 years of adulthood.

Model A

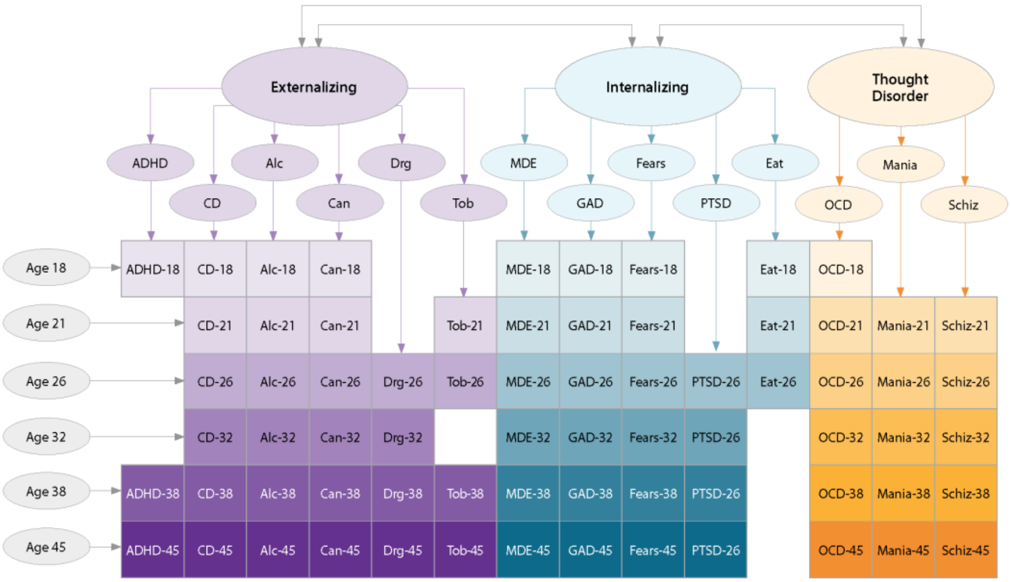

Our second model, the hierarchical or bifactor model tests the hypothesis that the symptom measures reflect both General Psychopathology and three narrower styles of psychopathology. General Psychopathology (labeled *p* in the figure below) is represented by a factor that directly influences all of the diagnostic symptom factors. In addition, styles of psychopathology are represented by three factors, each of which influences a smaller subset of the symptom items. For example, alcohol symptoms load jointly on the General Psychopathology factor and on the Externalizing style factor. The specific factors represent the constructs of Externalizing, Internalizing, and Thought Disorder over and above General Psychopathology.

Model B

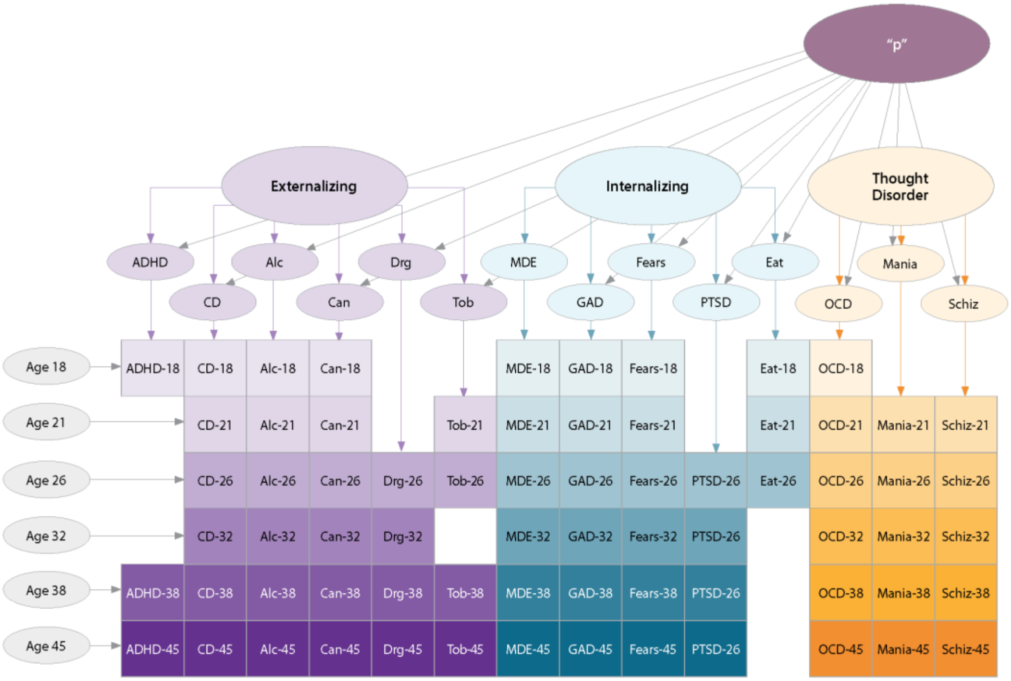

Model B had a Heywood case, an estimated variance that was negative for one of the lower-order disorder/symptom factors (specifically, mania), suggesting this was not a valid model. Inspection of the results revealed the source of the convergence problem. Specifically, the Thought Disorder factor was subsumed in *p*; that is, in the hierarchical model, symptoms of OCD, mania, and schizophrenia loaded very highly on *p*, but unlike symptoms of Externalizing and Internalizing, they could not form a separate Thought Disorder factor independently of *p*. We respecified the model accordingly, depicted in Model B′ below. This model fit the data well: χ^2(2457, N=1,000) = 3695.364, CFI = .949, TLI = .945, RMSEA = .022, 90% CI [.021, .024]. As shown in the Table on page 12, loadings on the General factor (*p*) were all positive, generally high (all ps < .001), and averaged .612; the highest standardized loadings were for mania (.976), schizophrenia (.865), PTSD (.860), and OCD (.772).

<u>Model B’</u>

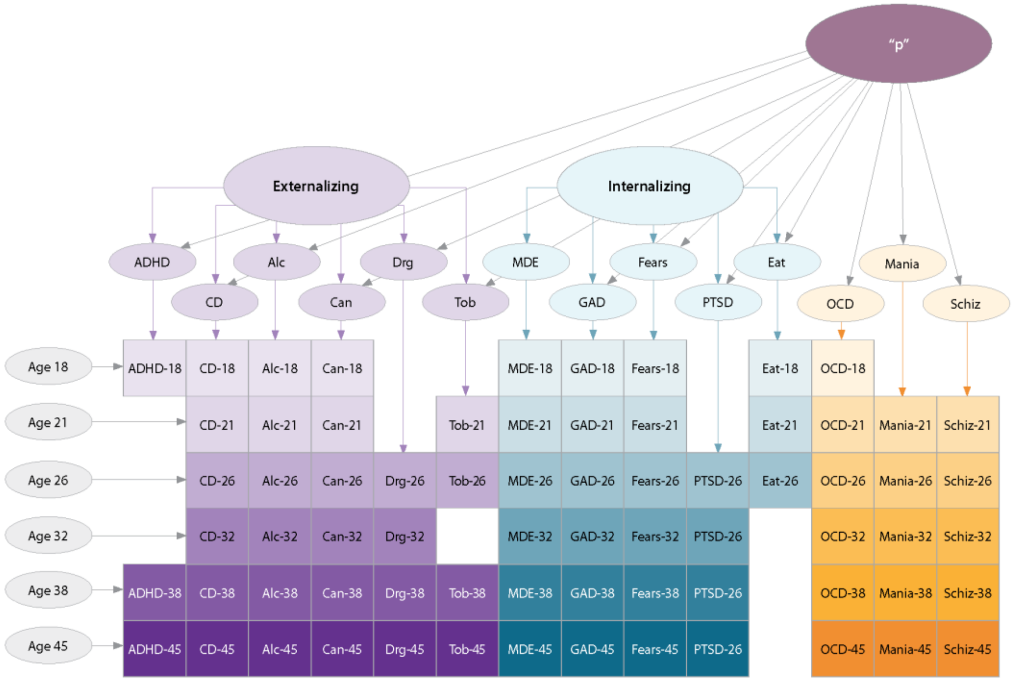

© 2020 Caspi A et al. *JAMA Network Open.*

The next Figure shows that the *p* factor (scaled to M = 100, SD = 15) captures how cohort members differ from each other in the variety and persistence of many different kinds of disorders over the adult life course. Cohort members with higher *p* scores experienced a greater variety of psychiatric disorders from early adolescence to midlife (r=.76 [95% CI: 0.74,0.79], p < .001).

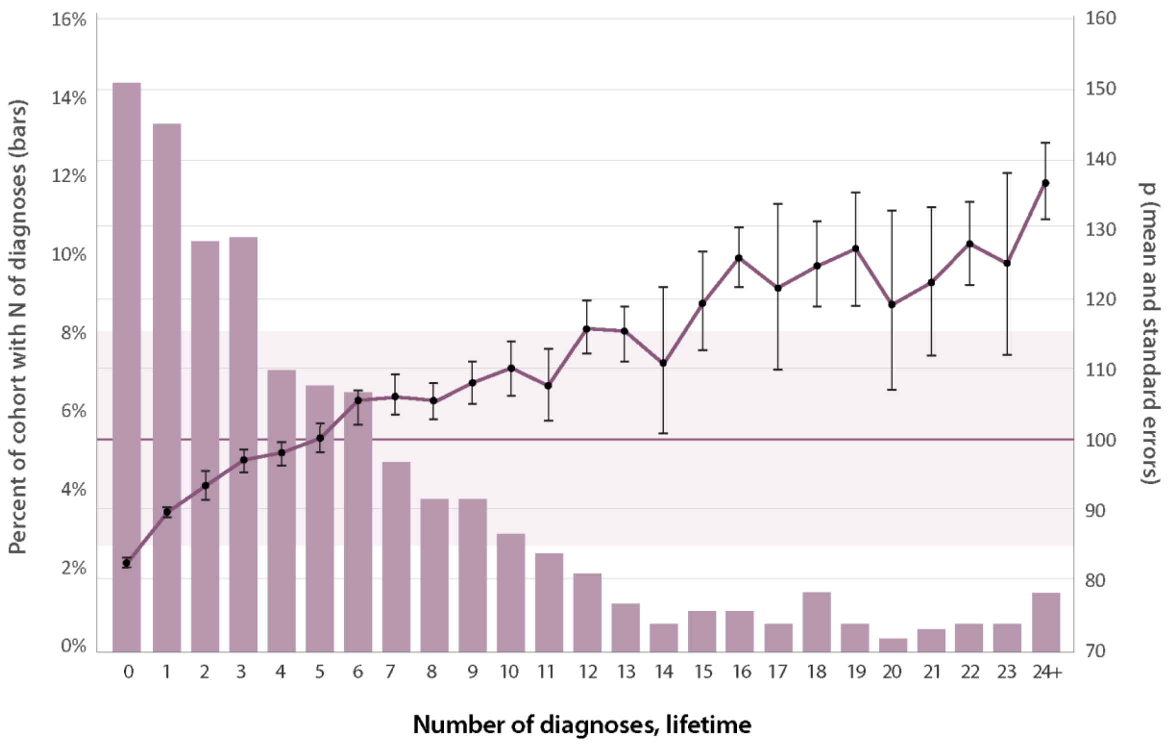

© 2020 Caspi A et al. *JAMA Network Open.*

The next table shows the standardized factor loadings for models of the structure of psychopathology.

	Model A: Correlated Factors			Model B': Bifactor Model with *p*		
	**Externalizing**	**Internalizing**	**Thought Disorder**	**p-factor**	**Externalizing**	**Internalizing**
**Model Fit Statistics**						
Chi-Square (WLSMV)	4082.230			3695.364		
Degrees of Freedom	2465			2457		
Comparative Fit Index	0.933			0.949		
Tucker-Lewis Index	0.929			0.945		
RMSEA [90% CI]	0.026 [0.024, 0.027]			0.022 [0.021, 0.024]		
**Standardized factor loadings**						
ADHD	0.567			0.595	0.121	
Alcohol	0.651			0.300	0.622	
Cannabis	0.831			0.369	0.850	
Hard drugs	0.845			0.466	0.694	
Tobacco	0.675			0.450	0.468	
Conduct disorder	0.888			0.504	0.714	
Major depression		0.968		0.768		0.587
Generalized anxiety		0.892		0.686		0.642
Fears/phobias		0.717		0.582		0.424
Eating disorder		0.499		0.377		0.374
PTSD		0.994		0.860		0.351
OCD			0.739	0.772		
Mania			0.955	0.976		
Schizophrenia			0.838	0.865		
**Factor Correlations**						
Externalizing		0.420	0.622			
Internalizing			0.847			

© 2020 Caspi A et al. *JAMA Network Open.*

eAppendix 5. Prevalence of Mental Disorder in the Dunedin Study

The figure shows the proportion of Dunedin cohort members meeting criteria for at least one psychiatric disorder in the preceding 12 months at each assessment phase, from early adolescence to midlife. Of 1037 original participants (53[51.6%] male), 1013 had mental-health data. At age 11-15 years, 35% (346/975) met criteria for a mental disorder, 50% (473/941) at age 18, 51% (489/961) at 21, 48% (472/977) at 26, 46% (444/969) at 32, 45% (429/955) at 38, and 44% (407/927) at 45. Cumulatively, by age 45, 86% (869/1013) of the cohort met criteria for at least one disorder.

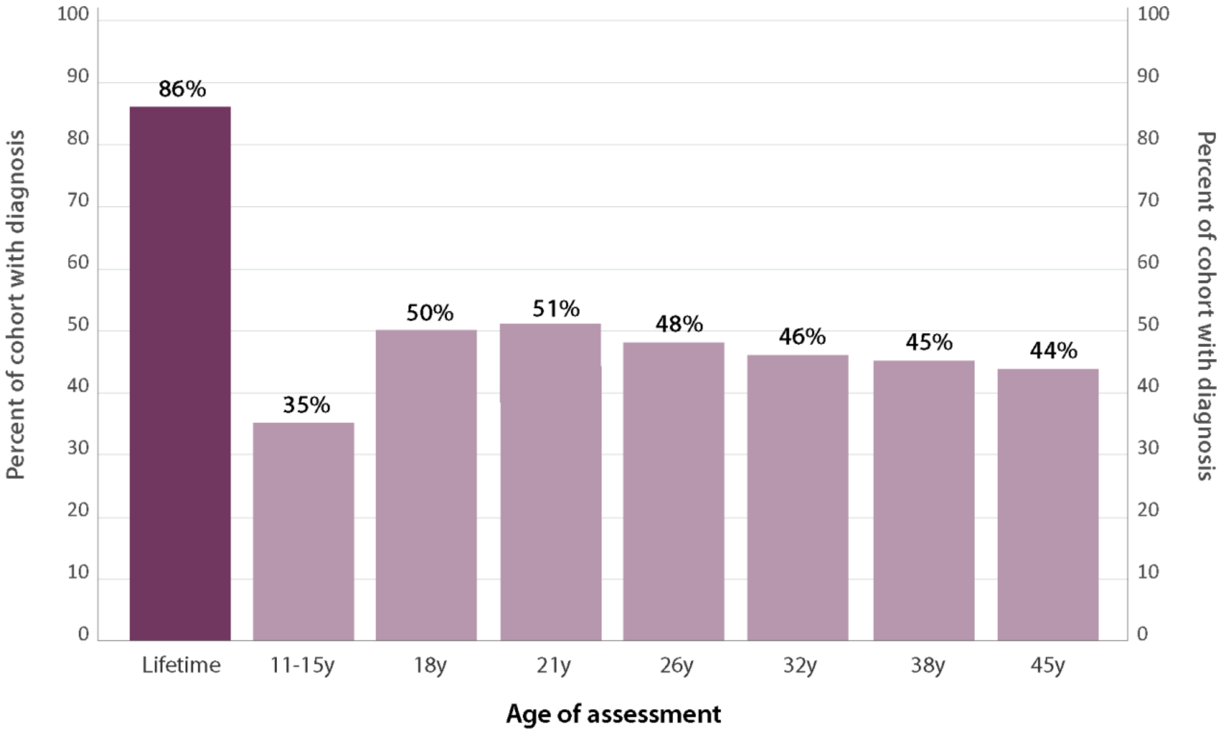

The high lifetime diagnosis rates in the Dunedin Study may come as a surprise to some readers. But, in fact, these rates are in line with data from other epidemiological studies, and with rates reported in other prospective-longitudinal studies. Specifically, multiple longitudinal-epidemiological studies from different countries converge on (a) finding that by age 15-16, approximately 35% of children meet criteria for a mental disorder and (b) that up to midlife the vast majority of people will have experienced a mental disorder in their lifetime.

Costello et al. [29] reported the cumulative prevalence of child and adolescent psychiatric disorder in a cohort of 1,420 North Carolina children who were first assessed when they were 9 to 13 years old and assessed annually thereafter. By age 16, 36.7% had received research diagnoses of at least one psychiatric disorder. In a cohort of 447 children from two upstate New York counties who were assessed for psychiatric disorder when they were 9 to 14 years of age and again at 12 to 16 years [30] 39% had been diagnosed with at least one psychiatric disorder by age 16. Among Dunedin Study participants, who were assessed for psychiatric disorder at 11, 13, and 15 years of age, we find that 35.4% met criteria for at least one psychiatric disorder by age 15. The consistency in these estimates of the cumulative prevalence of child and adolescent psychiatric disorder is striking given that the samples come from two countries (the United States and New Zealand), two regions of the United States (the rural south versus northeastern United States), and involve different historical cohorts of children (born in the 1960s and 1970s in New York and New Zealand and born in the 1980s in North Carolina).

By midlife, lifetime rates continue to accumulate. Prospective studies support the contention that retrospective and single-wave, cross-sectional studies underestimate the burden of disease in the population over time. As shown

© 2020 Caspi A et al. *JAMA Network Open.*

below, the lifetime rates that we report are in keeping with rates reported in all other prospective-longitudinal studies. First, we show data from studies of Scandinavian national registries which report that the lifetime prevalence of registered mental-disorder treatment is 33%. However, because many people with disorder are not treated, this is a lower bound. Second, we show data from cross-sectional surveys, such as the U.S. National Comorbidity Survey (NCS-R), that ask people to report retrospectively about their lifetime experience with mental disorders. These estimate lifetime prevalence near 50%. However, individuals with disorders resulting in homelessness, institutionalization, and survey refusal are missed in such surveys, and respondents' retrospective reports are documented to be biased by recall failure. Thus, 50% is an undercount. Third, we show data from the Dunedin Study and four other prospective birth cohort studies. These studies, begun decades ago, count cases irrespective of treatment, minimize recall failure, and gradually build participants' trust; these studies report that the vast majority of people experience a mental disorder at some point in their [31]. (Descriptions of the studies reported here appear on the next page.)

Study Design	N	Age Range	No. of Assessments	Length of Follow-up
**NATIONAL REGISTER**				
Danish Registry Data, males	5.6 million	0-100	–	–
Danish Registry Data, females	5.6 million	0-100	–	–
**RETROSPECTIVE SURVEY**				
National Comorbidity Survey	8098	15-54	1	–
National Comorbidity Survey Replication	9282	18-60	1	–
**PROSECTIVE COHORT**				
Great Smoky Mountain Cohort	1420	9-14 at baseline	up to 9	12 years
Oregon Adolescent Depression Project	816	16 at baseline	4	14 years
Zurich Cohort	591	19-20 at baseline	7	30 years
Christchurch Study	1041	15 at baseline	7	20 years
Dunedin Study	1013	11 at baseline	9	35 years

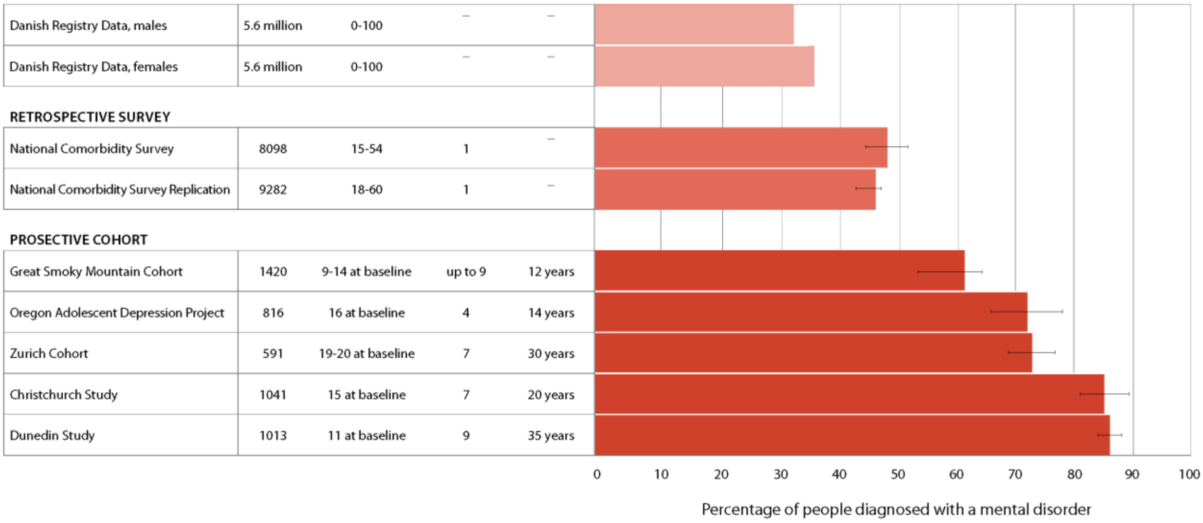

© 2020 Caspi A et al. *JAMA Network Open.*

Data sources for eAppendix 5:

Danish Registry Data. All Danish residents (*N* = approx. 5.6 million of each sex). Individuals were classified with a mental disorder if they had been admitted to a psychiatric hospital, received outpatient psychiatric care, or visited a psychiatric emergency unit [32].

National Comorbity Survey (NCS). Stratified, multistage area probability sample of persons aged 15 to 54 in the noninstitutionalized civilian population in the 48 coterminous United States (*N*=8098). Interviews were conducted with the Composite International Diagnostic Interview (CIDI).

National Comorbity Survey Replication (NCS-R). Nationally-representative sample of English-speaking household residents aged 18 years or older in the 48 coterminous United States (*N*=9282). Interviews were conducted as part of the World Mental Health Survey Initiative using the World Health Organization Composite International Diagnostic Interview (WMH-CIDI) [33].

Great Smoky Mountains Cohort. A representative sample of three cohorts of children ages 9, 11, and 13 years on intake from 11 counties in western North Carolina (N=1420). Interviews were conducted with the Child and Adolescent Psychiatric Assessment (CAPA) and the Young Adult Psychiatric Assessment (YAPA) [34].

Oregon Adolescent Depression Project. Cohort of high school students randomly selected from nine high schools in western Oregon (*N*=816). Interviews were conducted with the Schedule for Affective Disorders and Schizophrenia for School-Age Children (K-SADS), Longitudinal Interval Follow-Up Evaluation (LIFE), and Structured Clinical Interview for DSM-IV (SCID) [35].

The Zurich Cohort Study of Young Adults. Community-based cohort of 4,547 people aged 19-20 from Zurich Switzerland. A stratified subsample was selected for interview, with two-thirds consisting of high scorers on the global severity index of the SCL-90-R (*N* = 591). Interviews were conducted with Structured Psychopathological Interview and Rating of the Social Consequences of Psychological Disturbances for Epidemiology (SPIKE) [36].

The Christchurch Health and Development Study**.** Christchurch New Zealand birth cohort, born 1977 (N=1265). Interviews were conducted with the Diagnostic Interview Schedule for Children (DISC-C) and Composite International Diagnostic Interview (CIDI). Data provided by Dr. L.J. Horwood, October 7th, 2015.

Data from the Dunedin Multidisciplinary Health & Human Development Study, as described in the Main Article.

© 2020 Caspi A et al. *JAMA Network Open.*

eAppendix 6. Does Anyone Have Just One Exclusive Diagnosis?

The table provides the data graphed in Figure 3 of the Main Article. Panel A provides information about participants who were ever diagnosed by the Study with a mental disorder (N=869). Panel B restricts the analysis to participants who received inpatient mental-health services (N=83).

**Panel A.**	**Internalizing**		**Externalizing**		**Thought Disorder**	
	**N**	**% (95% CI)**	**N**	**% (95% CI)**	**N**	**% (95% CI)**
Any diagnosis	712	70.3% (67.4%, 73.1%)	625	61.7% (58.7%, 64.7%)	177	17.7% (14.8%, 20.1%)
Comorbid outside diagnostic family	503	70.6% (67.2%, 74.1%)	478	76.5% (73.1%, 79.9%)	174	98.3% (96.1%, 100.0%)
Comorbid within diagnostic family	113	15.9% (13.1%, 18.6%)	67	10.7% (8.2%, 13.2%)	0	0.0% (0.0%, 0.3%)
Single diagnosis within diagnostic family	96	13.5% (10.9%, 16.1%)	80	12.8% (10.1%, 15.5%)	3	1.7% (0.0%, 3.9%)

**Panel B.**	**Internalizing**		**Externalizing**		**Thought Disorder**	
	**N**	**% (95% CI)**	**N**	**% (95% CI)**	**N**	**% (95% CI)**
Any diagnosis	74	89.2% (81.9%, 96.4%)	70	84.3% (75.9%, 92.8%)	41	49.4% (38.0%, 60.8%)
Comorbid outside diagnostic family	68	91.9% (85.0%, 98.8%)	62	88.6% (80.4%, 96.7%)	41	100.0% (98.8%, 100.0%)
Comorbid within diagnostic family	5	6.8% (0.4%, 13.2%)	6	8.6% (1.3%, 15.8%)	0	0.0% (0.0%, 1.2%)
Single diagnosis within diagnostic family	1	1.4% (0.0%, 4.7%)	2	2.9% (0.0%, 7.5%)	0	0.0% (0.0%, 1.2%)

© 2020 Caspi A et al. *JAMA Network Open.*

eAppendix 7. Cross-Sectional Comorbidity.
The Venn diagrams show the overlap, at each assessment phase, between disorders grouped into three higher-order disorder-family categories: Internalizing, Externalizing, and Thought disorders. Each square represents 1% of the diagnosed Study members at each assessment phase.

Ages 11-15

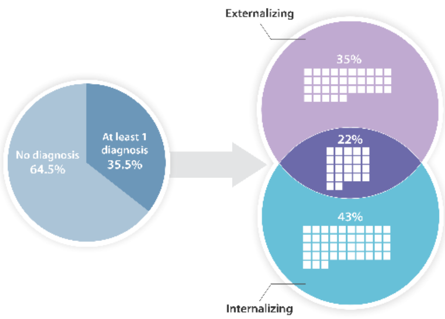

Age 18

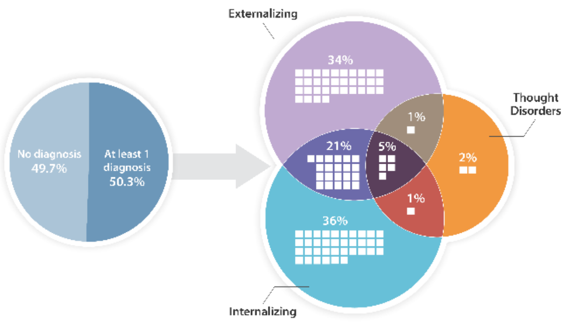

Age 21

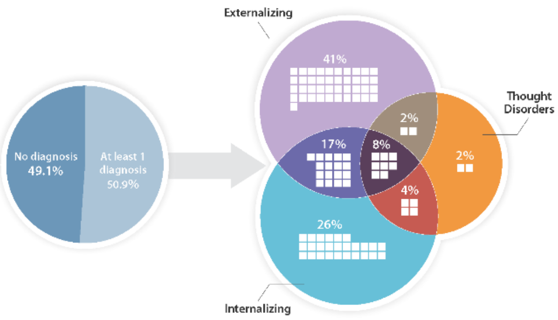

Age 26

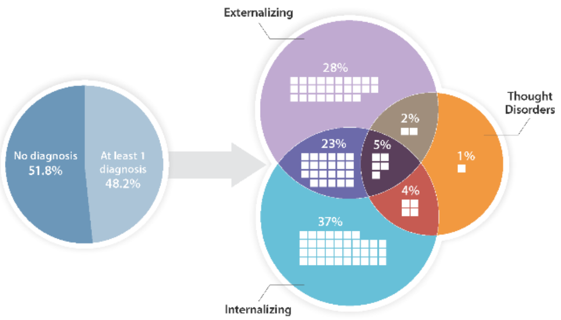

Age 32

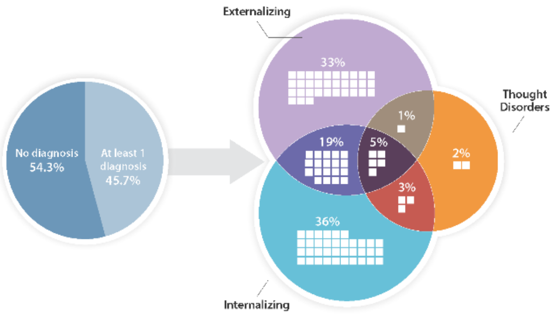

Age 38

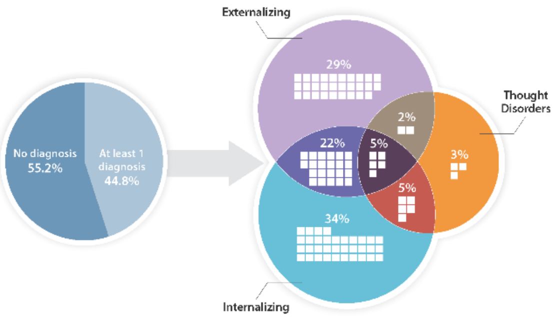

Age 45

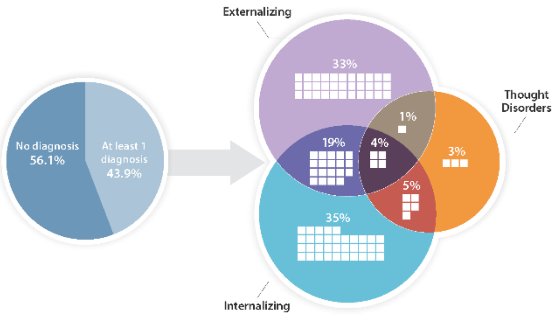

© 2020 Caspi A et al. *JAMA Network Open.*

The Table shows that Internalizing, Externalizing, and Thought disorders overlapped to a significant degree at all assessment phases. The Table shows the odds of meeting diagnostic criteria for a second disorder-family given meeting criteria for one disorder-family.

	**Internalizing & Externalizing**		**Internalizing & Thought Disorder**		**Externalizing & Thought Disorder**	
	**OR**	**95% CI**	**OR**	**95% CI**	**OR**	**95% CI**
Age 11/15	2.66	[1.89, 3.72]				
Age 18	2.02	[1.51, 2.70]	4.80	[2.50, 9.22]	4.59	[2.41, 8.73]
Age 21	1.81	[1.35, 2.41]	10.44	[6.02, 18.10]	3.15	[1.95, 5.07]
Age 26	2.50	[1.87, 3.35]	6.66	[3.50, 12.67]	3.66	[2.80, 6.42]
Age 32	2.18	[1.61, 2.94]	6.67	[3.60, 12.36]	4.04	[2.28, 7.14]
Age 38	2.81	[2.08, 3.81]	5.56	[3.20, 9.66]	2.54	[1.51, 4.29]
Age 45	2.17	[1.58, 2.99]	6.10	[3.40, 10.94]	1.81	[1.02, 3.20]

© 2020 Caspi A et al. *JAMA Network Open.*

eAppendix 8. Sequential Comorbidity

The first figure summarizes the sequential comorbidity of Internalizing, Externalizing, and Thought disorders. Participants with a disorder in any of the three diagnostic families at one specific age were at significantly higher risk for both other diagnostic families at subsequent ages. The Risk Ratios in black depict the continuity of the same disorders (e.g., "What is the risk of people with an Internalizing disorder at age 15 or at age 18, or at age 21, etc., presenting with a subsequent Internalizing disorder at later phases?"). The Risk Ratios in red depict sequential comorbidity (e.g., "What is the risk of people with an Internalizing disorder at age 15, or at age 18, or at age 21, etc., presenting with a subsequent Externalizing disorder at later phases?"). Average risk ratios across ages were calculated with a Generalized Estimating Equation (GEE) that nested individuals within time.

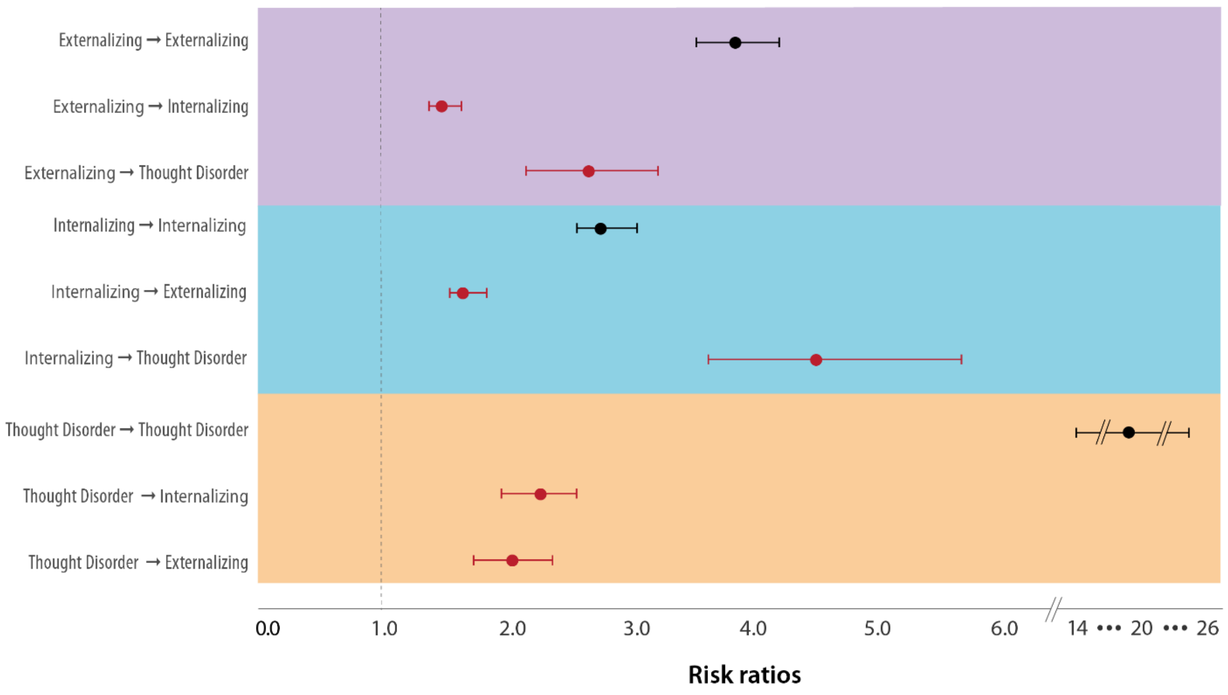

© 2020 Caspi A et al. *JAMA Network Open.*

The next figure shows the risk of presenting with a specific disorder at subsequent assessment waves given a specific disorder at an earlier assessment wave. The Risk Ratios on the diagonal depict the continuity of the same disorder; the off-diagonal Risk Ratios depict sequential comorbidity from the row diagnoses to the column diagnoses. Average Risk Ratios across assessment phases were calculated using Generalized Estimating Equations (GEE) that nested individuals within time. The overall impression in this figure is one of a uniform positive manifold: Individuals who meet criteria for one disorder are significantly more likely to subsequently meet criteria for the same disorder (along the diagonal) but also different disorders. Of the 196 risk ratios estimated, 183 (93%) were positive (only four risk ratios were <= 1.0 and nine could not be estimated given that models would not converge; these nine mostly involved eating disorders and mania, which had the lowest prevalence rates in the Study). The figure makes clear that longitudinal "cross-family" patterns are not confined to particular pairings, but are ubiquitous.

		To Subsequent Diagnosis																	
	**From Earlier Diagnosis:**	**1**	**2**	**3**	**4**	**5**	**6**		**7**	**8**	**9**	**10**	**11**		**12**	**13**	**14**	
**1**	ADHD	4.1	6.0	1.6	1.4	4.5	3.4		1.9	1.6	1.6	1.8	2.2		1.8		2.5	Not Observed (n = 9)
**2**	Conduct Disorder	3.4	17.6	2.5	2.7	5.9	7.8		1.1	1.6	1.5	0.8	2.2		1.6	2.0	4.7	< 1.0 (n = 4)
**3**	Alcohol Dependence	2.2	6.3	3.6	2.1	3.5	3.6		1.3	1.5	1.2	1.5	1.6		1.6	1.2	2.7	1.0 - < 2.0 (n = 67)
**4**	Tobacco Dependence	1.8	4.0	2.0	5.0	3.7	4.2		1.3	1.5	1.5	2.5	1.9		2.1	1.4	3.9	2.0 - < 3.0 (n = 53)
**5**	Cannabis Dependence	2.4	11.3	2.4	3.0	11.2	8.0		1.1	1.5	1.1	2.8	2.1		1.9	1.6	3.0	3.0 - < 4.0 (n = 25)
**6**	Drug Dependence	4.4	24.3	2.5	3.2	9.2	27.2		1.0	2.3	1.2		2.4		1.4	5.1	3.6	4.0 - < 5.0 (n = 13)
																		5.0 - < 6.0 (n = 5)
**7**	Anxiety	1.8	2.5	1.5	1.5	1.8	2.6		3.0	2.1	1.9		3.2		3.5	1.7	3.8	>= 6.0 (n = 20)
**8**	Depression	1.7	2.2	1.5	1.7	1.7	2.1		2.5	2.1	1.8	2.8	3.2		2.3	2.5	4.9	
**9**	Fears	1.6	1.7	1.1	1.5	1.5	2.1		2.5	1.8	2.8	2.7	3.3		3.6	1.2	3.1	
**10**	Eating Disorder	1.9	3.7	1.0	1.3	1.9	2.9		1.2	1.4	1.2	16.5	1.9		3.5	6.9	4.6	
**11**	PTSD	1.6	6.3	1.3	1.8	2.8	4.5		2.3	2.3	1.7		5.2		2.0		6.8	
**12**	OCD	1.7	2.8	1.4	1.5	2.2	2.9		3.3	2.2	2.3	3.2	3.6		7.0	3.0	6.7	
**13**	Mania			0.9	0.6	2.1	3.1		4.0	1.7	1.7		4.1		2.7	161.3	4.3	
**14**	Schizophrenia		10.0	2.2	2.7	2.6	4.4		2.3	2.4	2.1	2.9	7.0		6.0	3.0	185.5	

© 2020 Caspi A et al. *JAMA Network Open.*

Does unreliability of diagnoses create artefactual shifting among disorders? We further evaluated the impact of unreliability on sequential comorbidity. The test-retest reliability kappas in the Dunedin Study (based on N=60, retest interval average 60 days) for three groups of common disorders are: Anxiety (.49), depression (.73), and substance dependence (.88). These compare favorably with published estimates of retest reliabilities in structured psychiatric diagnostic interviews [37]. For diagnoses of schizophrenia and mania, we have had independent reviews by clinicians who achieved interrater agreement on cases [6]. Unreliability tends to be magnified in diagnostic data, where subthresholds are ignored. For example, a person with X number of symptoms is said to meet diagnostic criteria, but a person with X-1 does not, and such qualitative thresholds contribute to apparent unreliability. What impact does unreliability have? Unreliability in measurement underestimates associations between different disorders over time. To document this, we re-estimated our analyses which show longitudinal shifting of mental disorders across time. Our analyses of sequential comorbidity used GEE (General Estimating Equations) to estimate the risk that people with a specific disorder at one wave will present with a different disorder at subsequent waves, and to estimate the likelihood that this shifting occurs across Internalizing, Externalizing, and Thought disorders. We re-estimated these models using Latent Makov analyses (LM). LM is a form of error-in-variable model, which takes account of unreliability (one can think of it as a “correction” which tells us what the estimated association is expected to be if one could measure *X* and *Y* with perfect reliability of 100%). The table below shows the risk ratios from our original analysis (GEE) and the risk ratios from the Latent Markov Analysis (LM).

From earlier diagnosis:	To subsequent diagnosis:			
**Externalizing**	**Internalizing**		**Thought disorder**	
	GEE	*LM (models reliability)*	*GEE*	*LM (models reliability)*
	1.44 (1.32,1.57)	1.62 (1.34,1.90)	2.54 (2.04,3.17)	2.50(1.61,3.39)
**Internalizing**	**Externalizing**		**Thought disorder**	
	GEE	*LM*	*GEE*	*LM*
	1.59 (1.46,1.74)	1.80 (1.50,2.09)	4.47(3.52,5.68)	4.45 (2.94,5.95)
**Thought disorder**	**Externalizing**		**Internalizing**	
	GEE	*LM*	*GEE*	*LM*
	1.89 (1.63, 2.19)	2.61 (1.77,3.45)	2.13 (1.87,2.42)	3.45 (2.39,4.51)

As is apparent by comparing the GEE and Latent Markov estimates, unreliability does not affect the pattern of findings. The estimates were all significant in the GEE models, and they are, if anything, stronger in the Latent Markov models. We continue to see significant changes between disorders across repeated assessments years apart. This shows that diagnostic unreliability is not the culprit in why we observe shifting among different successive disorders over the life course. Moreover, whereas some of our analyses use qualitatitve diagnostic thresholds, our confirmatory factor analyses rely on quantitative symptom-level information (see eAppendix 4).

© 2020 Caspi A et al. *JAMA Network Open.*

eAppendix 9. Lifetime Comorbidity

The next figure contains information about lifetime comorbidity at the level of 14 individual disorders, rising from 32% of those with disorder by age 15 years to over 85% of those with disorder by age 45. The bottom row of each panel shows the proportion of Study members who met diagnostic criteria for a disorder; the second row of each panel shows the proportion of Study members who met diagnostic criteria for a second, different disorder; the third row of each panel shows the proportion of Study members who met criteria for a third, different disorder; and so on.

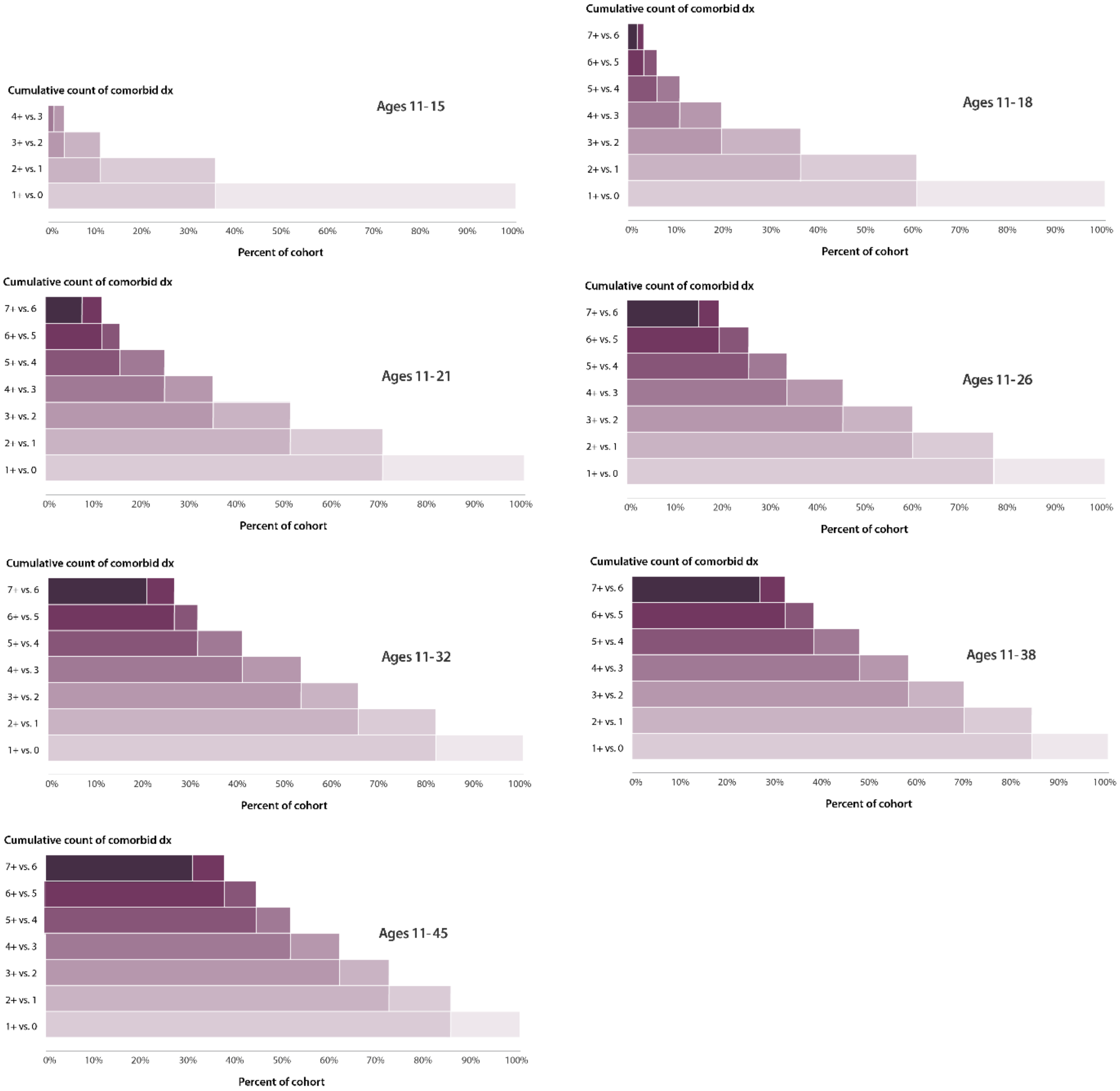

© 2020 Caspi A et al. *JAMA Network Open.*

Whereas the previous figure contains information about lifetime comorbidity at the level of specific disorders, the next figure contains information about lifetime comorbidity at the level of disorder-family categories: Internalizing, Externalizing, and Thought disorders.

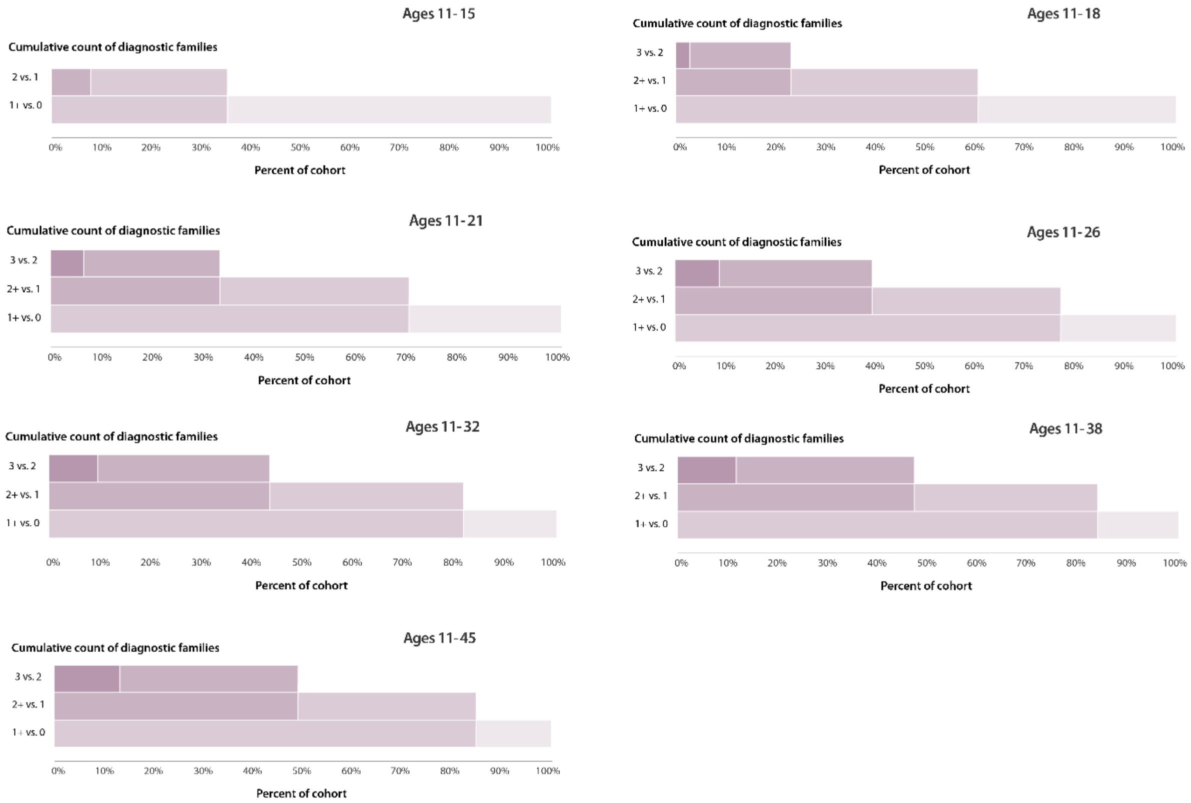

© 2020 Caspi A et al. *JAMA Network Open.*

eAppendix 10. The Ebb and Flow of Mental Disorders Among Participants Who Received Inpatient Mental-Health Services.

Over the course of the Study, 83 participants received inpatient services. The Sankey chart highlights the 83 inpatient mental-disorder life-histories embedded within the mental-disorder life-histories of the entire cohort, which are shown in gray background (see Figure 4 in the Main Article). (Note: it is possible to follow groups across contiguous assessments, not across the entire panel.)

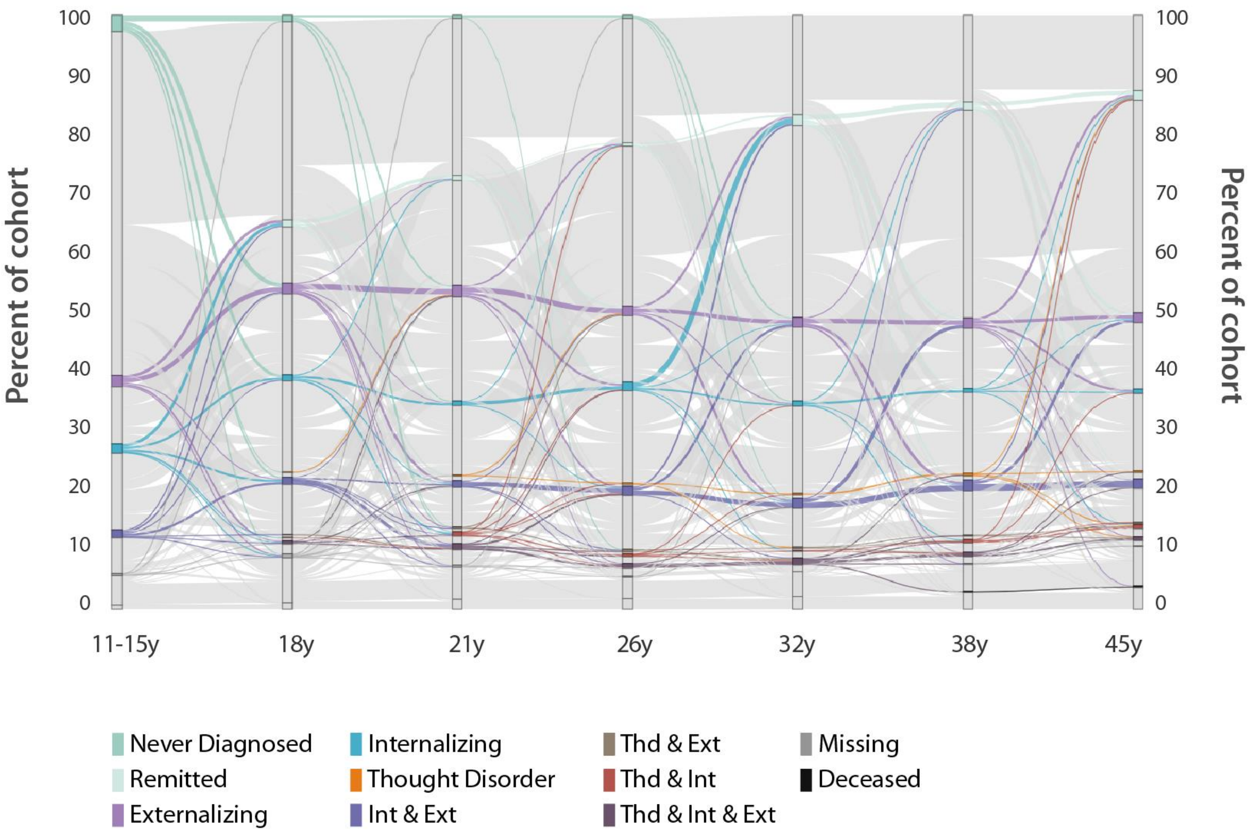

© 2020 Caspi A et al. *JAMA Network Open.*

eAppendix 11. Correlations Between Measures of Mental-Disorder Life-Histories and Measures of Brain Function From Childhood to Midlife.

Scatterplots A, B, and C show the raw data used to build the graphs in Figure 5 of the Main Article. Scatterplot A shows the correlation between p-factor scores and scores on age-3 brain health; scatterplot B shows the correlation between p-factor scores and measures of IQ change (adult[minus]child score); scatterplot C shows the correlation between p-factor scores and each participant's estimated brain age.

Scatterplot A: p-factor with age 3 brain health

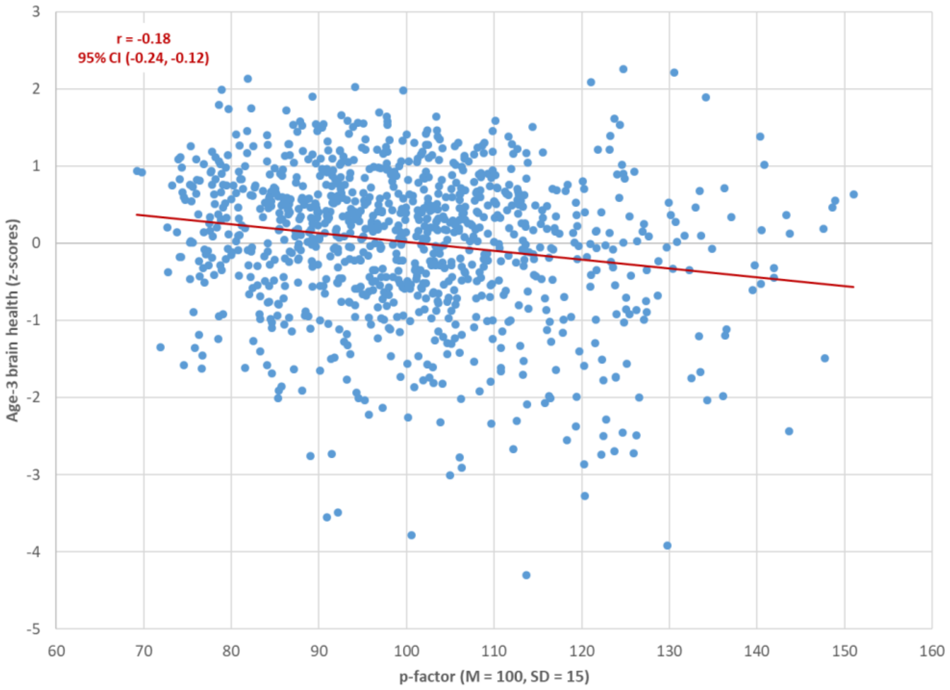

© 2020 Caspi A et al. *JAMA Network Open.*

Scatterplot B: p-factor with IQ change

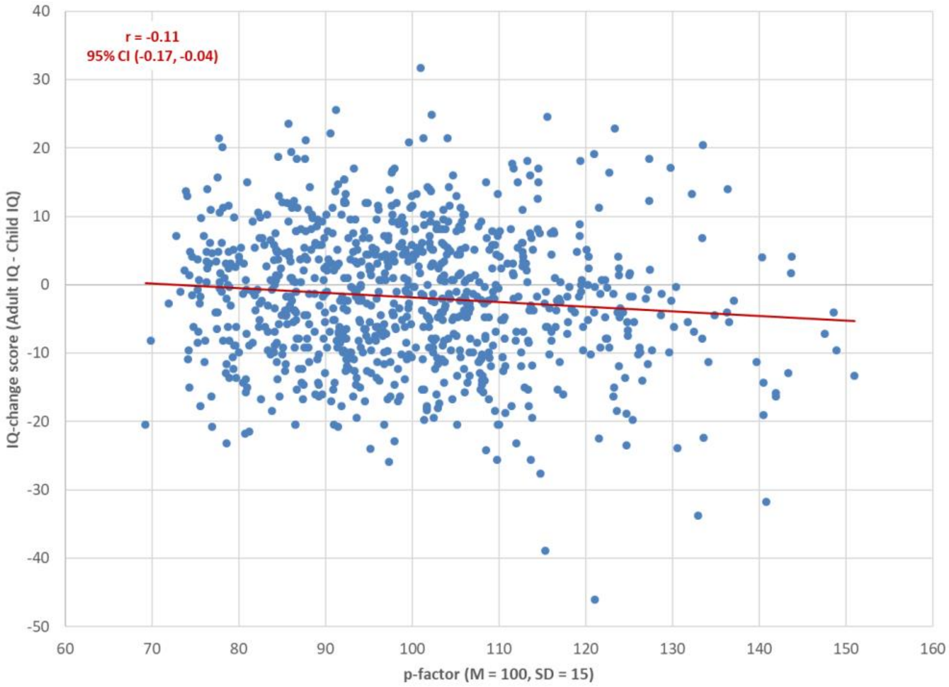

Scatterplot C: p-factor with age-45 brain age

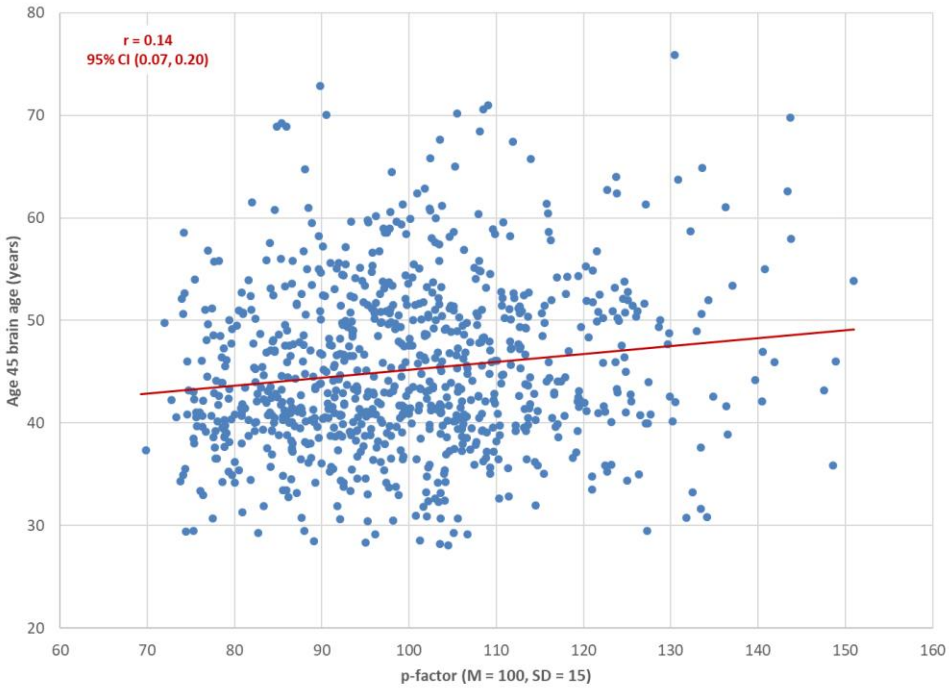

© 2020 Caspi A et al. *JAMA Network Open.*

The Main Article, and the scatterplots above, report the associations between p-factor scores and measures of brain function. These correlations are shown in the shaded column of the next table. The table also shows the correlations between age-of-onset of mental disorder, duration (number of assessment phases during which the Study member met diagnostic criteria for a disorder), and diversity (number of different types of disorder) and measures of brain function. The purpose of the table is to show how *p* summarizes three key developmental parameters of mental-disorder life-histories.

	**p-factor**	**Age of Onset**	**Duration**	**Diversity**
Child Brain Health	-0.18 (-0.24, -0.12)	0.09 (0.03, 0.15)	-0.12 (-0.18, -0.06)	-0.15 (-0.21, -0.09)
Child IQ	-0.19 (-0.25, -0.13)	0.11 (0.05, 0.17)	-0.14 (-0.20, -0.08)	-0.17 (-0.23, -0.11)
Adult IQ	-0.24 (-0.30, -0.18)	0.17 (0.11, 0.23)	-0.25 (-0.31, -0.19)	-0.27 (-0.33, -0.21)
IQ Decline	-0.11 (-0.17, -0.04)	0.09 (0.03, 0.16)	-0.14 (-0.20, -0.07)	-0.15 (-0.21, -0.08)
Brain Age	0.14 (0.07, 0.20)	-0.07 (-0.13, 0.00)	0.14 (0.07, 0.20)	0.15 (0.08, 0.21)

Note: Numbers represent Pearson r's and 95% confidence intervals.

The next table shows the correlations between the Internalizing, Externalizing, and Thought disorder factors and the measures of brain function. (The three psychopathology factors are derived from the Correlated Factor Model described in eAppendix 4.) The results show that each of the three psychopathology factors was antedated by age-3 brain dysfunction, accompanied by child-to-adult cognitive decline, and associated with older brain-age at midlife. There was no specificity. This nonspecificity is understandable in light of the life-history evidence that people's diagnosis changes frequently, which is summarized parsimoniously in *p*.

	**p-factor**	**Thought Disorders factor**	**Internalizing Factor**	**Externalizing factor**
Child Brain Health	-0.18 (-0.24, -0.12)	-0.17 (-0.23, -0.11)	-0.14 (-0.20, -0.07)	-0.10 (-0.16, -0.04)
Child IQ	-0.19 (-0.25, -0.13)	-0.17 (-0.23, -0.11)	-0.16 (-0.22, -0.10)	-0.08 (-0.14, -0.02)
Adult IQ	-0.24 (-0.30, -0.18)	-0.23 (-0.29, -0.17)	-0.18 (-0.24, -0.12)	-0.20 (-0.26, -0.14)
IQ Decline	-0.11 (-0.17, -0.04)	-0.11 (-0.17, -0.05)	-0.05 (-0.12, 0.01)	-0.18 (-0.24, -0.11)
Brain Age	0.14 (0.04, 0.20)	0.16 (0.10, 0.23)	0.17 (0.10, 0.23)	0.14 (0.07, 0.20)

Note: Numbers represent Pearson r's and 95% confidence intervals.

eAppendix 12. MPlus Syntax.

MPlus v8.3 syntax for correlated factors model.

```
TITLE:  Correlated Factor Model w/ Method Factor;

DATA:   FILE = symp_June2019.dat;

VARIABLE:
        NAMES ARE
            snum sex
            adhd18                          adhd38  adhd45
            adhd18b                         adhd38b adhd45b
                           ptsd26 ptsd32 ptsd38  ptsd45
            Alc18   Alc21  Alc26  Alc32  Alc38   Alc45
            Mar18   Mar21  Mar26  Mar32  Mar38   Mar45
            Drg26   Drg32  Drg38  Drg45
            smk21   smk26                smk38   smk45
            cd18    cd21   cd26   cd32   cd38    cd45
            mde18   mde21  mde26  mde32  mde38   mde45
            Gad18   Gad21  Gad26  Gad32  Gad38   Gad45
            Fear18  Fear21 Fear26 Fear32 Fear38  Fear45
            Anrx18  Anrx21 Anrx26
            Bul18   Bul21  Bul26
            OCD18   OCD21  OCD26  OCD32  OCD38   OCD45
            Man21   Man26  Man32  Man38  Man45
            scz21   scz26  scz32  scz38  scz45;

        MISSING
            ALL (9999);

        USEVARIABLES ARE
            adhd18b                              adhd38b adhd45b
                           ptsd26 ptsd32 ptsd38  ptsd45
            Alc18   Alc21  Alc26  Alc32  Alc38   Alc45
            Mar18   Mar21  Mar26  Mar32  Mar38   Mar45
            Drg26   Drg32  Drg38  Drg45
            smk21   smk26                smk38   smk45
            cd18    cd21   cd26   cd32   cd38    cd45
            mde18   mde21  mde26  mde32  mde38   mde45
            Gad18   Gad21  Gad26  Gad32  Gad38   Gad45
            Fear18  Fear21 Fear26 Fear32 Fear38  Fear45
            Anrx18  Anrx21 Anrx26
            Bul18   Bul21  Bul26
            OCD18   OCD21  OCD26  OCD32  OCD38   OCD45
            Man21   Man26  Man32  Man38  Man45
            scz21   scz26  scz32  scz38  scz45;

        CATEGORICAL ARE
                          ptsd26 ptsd32 ptsd38 ptsd45
            Alc18  Alc21  Alc26  Alc32  Alc38  Alc45
            Mar18  Mar21  Mar26  Mar32  Mar38  Mar45
                          Drg26  Drg32  Drg38  Drg45
                   smk21  smk26         smk38  smk45
            cd18   cd21   cd26   cd32   cd38   cd45
            mde18  mde21  mde26  mde32  mde38  mde45
            Gad18  Gad21  Gad26  Gad32  Gad38  Gad45
            Fear18 Fear21 Fear26 Fear32 Fear38 Fear45
            Anrx18 Anrx21 Anrx26
            Bul18  Bul21  Bul26
            OCD18  OCD21  OCD26  OCD32  OCD38  OCD45
                   Man21  Man26  Man32  Man38  Man45
                   scz21  scz26  scz32  scz38  scz45;

        IDVARIABLE IS
            snum;

ANALYSIS:

MODEL:
```

© 2020 Caspi A et al. *JAMA Network Open.*

```
! Define Mental Health factors across time;
adhd  BY adhd18b                    adhd38b adhd45b;
alc   BY Alc18   Alc21  Alc26  Alc32  Alc38   Alc45;
mar   BY Mar18   Mar21  Mar26  Mar32  Mar38   Mar45;
drg   BY                Drg26  Drg32  Drg38   Drg45;
smk   BY         Smk21  Smk26         Smk38   Smk45;
cd    BY CD18    CD21   CD26   CD32   CD38    CD45;

mde   BY Mde18   Mde21  Mde26  Mde32  Mde38   Mde45;
gad   BY Gad18   Gad21  Gad26  Gad32  Gad38   Gad45;
fear  BY Fear18  Fear21 Fear26 Fear32 Fear38  Fear45;
ptsd  BY                ptsd26 ptsd32 ptsd38  ptsd45;

ocd   BY OCD18  OCD21   OCD26  OCD32  OCD38   OCD45;
mania BY        Man21   Man26  Man32  Man38   Man45;
scz   BY        scz21   scz26  scz32  scz38   scz45;

eat   BY anrx18 anrx21 anrx26
         bul18  bul21  bul26;

! Define Externalizing, Internalizing & Thought Disorder factors;
ext BY alc* mar drg smk adhd CD;
int BY mde* gad ptsd eat fear;
thd BY ocd* mania scz;

! Standardize (scale) to Mean = 0 Var = 1;
[ext@0 int@0 thd@0];
 ext@1 int@1 thd@1;

! Correlations across measured symptom scales within a measurement period;
!    To account for measurement time anomalies;
!    These are uncorrelated with each other and uncorrelated with
!    psychopathology factors;

Age18 BY adhd18b* alc18 mar18 CD18 mde18 gad18 fear18 anrx18 bul18 ocd18;
Age21 BY alc21* mar21 smk21 CD21 mde21 gad21 fear21 anrx21 bul21 ocd21 man21
         scz21;
Age26 BY alc26* mar26 drg26 smk26 CD26 mde26 gad26 fear26 anrx26 bul21 ptsd26
         ocd26 man26 scz26;
Age32 BY alc32* mar32 drg32 CD32 mde32 gad32 fear32 ptsd32 ocd32  man32  scz32;
Age38 BY adhd38b* alc38 mar38 drg38 smk38 CD38 mde38 gad38 fear38 ptsd38 ocd38
         man38 scz38;
Age45 BY adhd45b* alc45 mar45 drg45 smk45 CD45 mde45 gad45 fear45 ptsd45 ocd45
         man45 scz45;

! Standardize (scale) to Mean = 0 Var = 1;
[age18@0 age21@0 age26@0 age32@0 age38@0 age45@0];
 age18@1 age21@1 age26@1 age32@1 age38@1 age45@1;

! Set correlations to 0;
age18 WITH age21@0 age26@0 age32@0 age38@0 age45@0;
age21 WITH age26@0 age32@0 age38@0 age45@0;
age26 WITH age32@0 age38@0 age45@0;
age32 WITH age38@0 age45@0;
age38 WITH age45@0;

ext WITH age18@0 age21@0 age26@0 age32@0 age38@0 age45@0;
int WITH age18@0 age21@0 age26@0 age32@0 age38@0 age45@0;
thd WITH age18@0 age21@0 age26@0 age32@0 age38@0 age45@0;

! Allow Externalizing, Internalizing, Thought Disorders factors to correlate;
ext WITH int thd;
int WITH thd;

OUTPUT: TECH1 TECH4 STANDARDIZED

SAVEDATA:    FILE = CF_JUNE2019.dat;
             SAVE = FSCORES;
```

© 2020 Caspi A et al. *JAMA Network Open.*

MPlus v8.3 syntax for Bifactor Model B'

```
TITLE:   Bi-Factor Model w/ Method Factor;

DATA:    FILE = symp_June2019.dat;

VARIABLE:
        NAMES ARE
            snum sex
            adhd18                      adhd38  adhd45
            adhd18b                     adhd38b adhd45b
                           ptsd26 ptsd32 ptsd38  ptsd45
            Alc18   Alc21  Alc26  Alc32  Alc38   Alc45
            Mar18   Mar21  Mar26  Mar32  Mar38   Mar45
            Drg26   Drg32  Drg38  Drg45
            smk21   smk26               smk38   smk45
            cd18    cd21   cd26   cd32   cd38    cd45
            mde18   mde21  mde26  mde32  mde38   mde45
            Gad18   Gad21  Gad26  Gad32  Gad38   Gad45
            Fear18  Fear21 Fear26 Fear32 Fear38  Fear45
            Anrx18  Anrx21 Anrx26
            Bul18   Bul21  Bul26
            OCD18   OCD21  OCD26  OCD32  OCD38   OCD45
            Man21   Man26  Man32  Man38  Man45
            scz21   scz26  scz32  scz38  scz45;

        MISSING
            ALL (9999);

        USEVARIABLES ARE
            adhd18b                    adhd38b adhd45b
                          ptsd26 ptsd32 ptsd38 ptsd45
            Alc18  Alc21  Alc26  Alc32  Alc38  Alc45
            Mar18  Mar21  Mar26  Mar32  Mar38  Mar45
                          Drg26  Drg32  Drg38  Drg45
                   smk21  smk26         smk38  smk45
            cd18   cd21   cd26   cd32   cd38   cd45
            mde18  mde21  mde26  mde32  mde38  mde45
            Gad18  Gad21  Gad26  Gad32  Gad38  Gad45
            Fear18 Fear21 Fear26 Fear32 Fear38 Fear45
            Anrx18 Anrx21 Anrx26
            Bul18  Bul21  Bul26
            OCD18  OCD21  OCD26  OCD32  OCD38  OCD45
                   Man21  Man26  Man32  Man38  Man45
                   scz21  scz26  scz32  scz38  scz45;

        CATEGORICAL ARE
                          ptsd26 ptsd32 ptsd38 ptsd45
            Alc18  Alc21  Alc26  Alc32  Alc38  Alc45
            Mar18  Mar21  Mar26  Mar32  Mar38  Mar45
                          Drg26  Drg32  Drg38  Drg45
                   smk21  smk26         smk38  smk45
            cd18   cd21   cd26   cd32   cd38   cd45
            mde18  mde21  mde26  mde32  mde38  mde45
            Gad18  Gad21  Gad26  Gad32  Gad38  Gad45
            Fear18 Fear21 Fear26 Fear32 Fear38 Fear45
            Anrx18 Anrx21 Anrx26
            Bul18  Bul21  Bul26
            OCD18  OCD21  OCD26  OCD32  OCD38  OCD45
                   Man21  Man26  Man32  Man38  Man45
                   scz21  scz26  scz32  scz38  scz45;

        IDVARIABLE IS
            snum;

ANALYSIS:

MODEL:
    ! Define Mental Health factors across time;
    adhd  BY adhd18b                      adhd38b adhd45b;
    alc   BY Alc18  Alc21  Alc26  Alc32  Alc38  Alc45;
```

© 2020 Caspi A et al. *JAMA Network Open.*

```
    mar   BY Mar18  Mar21  Mar26  Mar32  Mar38  Mar45;
    drg   BY               Drg26  Drg32  Drg38  Drg45;
    smk   BY        Smk21  Smk26         Smk38  Smk45;
    cd    BY CD18   CD21   CD26   CD32   CD38   CD45;

    mde   BY Mde18  Mde21  Mde26  Mde32  Mde38  Mde45;
    gad   BY Gad18  Gad21  Gad26  Gad32  Gad38  Gad45;
    fear  BY Fear18 Fear21 Fear26 Fear32 Fear38 Fear45;
    ptsd  BY               ptsd26 ptsd32 ptsd38 ptsd45;

    ocd   BY OCD18  OCD21  OCD26  OCD32  OCD38  OCD45;
    mania BY        Man21  Man26  Man32  Man38  Man45;
    scz   BY        scz21  scz26  scz32  scz38  scz45;

    eat   BY anrx18 anrx21 anrx26
             bul18  bul21  bul26;

    ! Define Externalizing & Internalizing factors;
    !    ... uncorrelated w/ "Little P";
    !    ... correlated with each other;
    ext  BY alc* adhd mar drg smk CD;
    int  BY mde* ptsd eat gad fear;

    ! Define "p-factor";
    !   ... uncorrelated with Externalizing & Internalizing;
    p BY adhd* alc mar drg smk CD mde gad fear ptsd eat ocd mania scz;

    ! Standardize (scale) to Mean = 0 Var = 1;
    [ext@0 int@0 p@0];
     ext@1 int@1 p@1;

    ! Correlations across measured symptom scales within a measurement period;
    !    To account for measurement time anomalies;
    !    These are uncorrelated with each other and uncorrelated with
    !    psychopathology factors;

    Age18 BY adhd18b* alc18 mar18 CD18 mde18 gad18 fear18 anrx18 bul18 ocd18;
    Age21 BY alc21* mar21 smk21 CD21 mde21 gad21 fear21 anrx21 bul21 ocd21 man21
             scz21;
    Age26 BY alc26* mar26 drg26 smk26 CD26 mde26 gad26 fear26 anrx26 bul21 ptsd26
             ocd26 man26 scz26;
    Age32 BY alc32* mar32 drg32 CD32 mde32 gad32 fear32 ptsd32 ocd32 man32 scz32;
    Age38 BY adhd38b* alc38 mar38 drg38 smk38 CD38 mde38 gad38 fear38 ptsd38 ocd38
             man38 scz38;
    Age45 BY adhd45b* alc45 mar45 drg45 smk45 CD45 mde45 gad45 fear45 ptsd45 ocd45
             man45 scz45;

    ! Standardize (scale) to Mean = 0 Var = 1;
    [age18@0 age21@0 age26@0 age32@0 age38@0 age45@0];
     age18@1 age21@1 age26@1 age32@1 age38@1 age45@1;

    ! Set correlations to 0;
    age18 WITH age21@0 age26@0 age32@0 age38@0 age45@0;
    age21 WITH age26@0 age32@0 age38@0 age45@0;
    age26 WITH age32@0 age38@0 age45@0;
    age32 WITH age38@0 age45@0;
    age38 WITH age45@0;

    ext WITH age18@0 age21@0 age26@0 age32@0 age38@0 age45@0;
    int WITH age18@0 age21@0 age26@0 age32@0 age38@0 age45@0;
    p   WITH age18@0 age21@0 age26@0 age32@0 age38@0 age45@0;

    p   WITH ext@0 int@0;
    ext WITH int@0;

OUTPUT: SAMPSTAT TECH1 TECH4 STANDARDIZED

SAVEDATA:   FILE = BF_17JUN2019.dat;
            SAVE = FSCORES;
            SAMPLE IS corrmat_June2019.dat;
```

© 2020 Caspi A et al. *JAMA Network Open.*

eReferences

1. Poulton R, Moffitt TE, Silva PA. The Dunedin Multidisciplinary Health and Development Study: overview of the first 40 years, with an eye to the future. *Soc Psychiatry Psychiatr Epidemiol.* 2015;50(5):679-693.
2. Richmond-Rakerd LS, D'Souza S, Andersen SH, et al. Clustering of health, crime and social-welfare inequality in 4 million citizens from two nations. *Nat Hum Behav.* 2020.
3. Caspi A, Moffitt TE, Thornton A, et al. The life history calendar: A research and clinical assessment method for collecting retrospective event-history data. *International Journal of Methods in Psychiatric Research.* 1996.
4. Robins LN, Cottler L, Bucholz K, Compton W. *Diagnostic interview schedule for DSM-IV.* St. Louis, MO: Washington University Press; 1995.
5. Robins LN, Helzer JE, Croughan J, Ratcliff KS. National Institute of Mental Health Diagnostic Interview Schedule: Its History, Characteristics, and Validity. *Archives of General Psychiatry.* 1981;38(4):381-389.
6. Meier MH, Caspi A, Reichenberg A, et al. Neuropsychological decline in schizophrenia from the premorbid to the postonset period: evidence from a population-representative longitudinal study. *Am J Psychiatry.* 2014;171(1):91-101.
7. APA. *American Psychological Association: Diagnostic and Statistical Manual of Mental Disorders.* Third ed. Washington, DC: American Psychiatric Association; 1980.
8. APA. *American Psychological Association: Diagnostic and Statistical Manual of Mental Disorders.* Revised Third ed. Washington, DC: American Psychiatric Association; 1987.
9. APA. *American Psychological Association: Diagnostic and Statistical Manual of Mental Disorders.* Fourth ed. Washington, DC: American Psychiatric Association; 1994.
10. APA. *American Psychological Association: Diagnostic and Statistical Manual of Mental Disorders.* Fifth ed. Washington, DC: American Psychiatric Association 2013.
11. Poulton R, Caspi A, Moffitt TE, Cannon M, Murray R, Harrington H. Children's self-reported psychotic symptoms and adult schizophreniform disorder: a 15-year longitudinal study. *Arch Gen Psychiatry.* 2000;57(11):1053-1058.
12. Fullana MA, Mataix-Cols D, Caspi A, et al. Obsessions and compulsions in the community: prevalence, interference, help-seeking, developmental stability, and co-occurring psychiatric conditions. *Am J Psychiatry.* 2009;166(3):329-336.
13. Caspi A, Houts RM, Belsky DW, et al. The p Factor:One General Psychopathology Factor in the Structure of Psychiatric Disorders? *Clinical Psychological Science.* 2014;2(2):119-137.
14. Touwen BCL, Prechtl HFR. The neurological examination of the child with minor nervous dysfunction. *Clinics in developmental medicine, no 38.* 1970:7 , vi, 105 p.
15. Dunn L. *The Peabody Picture Vocabulary Test.* Minneapolis: American Guidance Service; 1995.
16. Bayley N. *The Bayley Scale of Infant Development.* New York: Psychological Corp.; 1969.
17. Caspi A, Henry B, McGee RO, Moffitt T, Silva PA. Temperamental origins of child and adolescent behavior problems: from age three to age fifteen. *Child Dev.* 1995;66(1):55-68.
18. Wechsler D. *Wechsler Intelligence Scale for Children: WISC-R.* Psychological Corporation; 1974.
19. Moffitt TE, Arseneault L, Belsky D, et al. A gradient of childhood self-control predicts health, wealth, and public safety. *Proc Natl Acad Sci U S A.* 2011;108(7):2693-2698.
20. Wechsler D. *Wechsler Adult Intelligence Scale: WAIS-IV: Technical and Interpretive Manual.* Pearson; 2008.
21. Tucker-Drob EM, Brandmaier AM, Lindenberger U. Coupled cognitive changes in adulthood: A meta-analysis. *Psychol Bull.* 2019;145(3):273-301.
22. Liem F, Varoquaux G, Kynast J, et al. Predicting brain-age from multimodal imaging data captures cognitive impairment. *Neuroimage.* 2017;148:179-188.
23. Liang H, Zhang F, Niu X. Investigating systematic bias in brain age estimation with application to post-traumatic stress disorders. *Hum Brain Mapp.* 2019;40(11):3143-3152.

© 2020 Caspi A et al. *JAMA Network Open.*

24. Heatherton TF, Kozlowski LT, Frecker RC, Fagerstrom KO. The Fagerstrom Test for Nicotine Dependence: a revision of the Fagerstrom Tolerance Questionnaire. *Br J Addict.* 1991;86(9):1119-1127.
25. Caspi A, Moffitt T. All for One and One for All: Mental Disorders in One Dimension. *American Journal of Psychiatry.* 2018;175(9):831-844.
26. Muthen LK, Muthen BO. *Mplus User's Guide.* Eighth ed. Los Angeles, CA: Muthen & Muthen; 1998-2017.
27. Asparouhov T, Muthen BO. Weighted least squares estimation with missing data. http://www.statmodel.com/download/GstrucMissingRevision.pdf. Published 2010. Accessed.
28. Bollen KA, Curran PJ. Latent Curve Models A Structural Equation Perspective Introduction. *Wiley Ser Probab St.* 2006:1-15.
29. Costello EJ, Mustillo S, Erkanli A, Keeler G, Angold A. Prevalence and development of psychiatric disorders in childhood and adolescence. *Arch Gen Psychiatry.* 2003;60(8):837-844.
30. Cohen P, Cohen J, Kasen S, et al. An epidemiological study of disorders in late childhood and adolescence--I. Age- and gender-specific prevalence. *J Child Psychol Psychiatry.* 1993;34(6):851-867.
31. Schaefer JD, Caspi A, Belsky DW, et al. Enduring mental health: Prevalence and prediction. *J Abnorm Psychol.* 2017;126(2):212-224.
32. Pedersen CB, Mors O, Bertelsen A, et al. A Comprehensive Nationwide Study of the Incidence Rate and Lifetime Risk for Treated Mental DisordersRate of and Risk for Mental Disorders in DenmarkRate of and Risk for Mental Disorders in Denmark. *JAMA Psychiatry.* 2014;71(5):573-581.
33. Kessler RC, Berglund P, Demler O, Jin R, Merikangas KR, Walters EE. Lifetime Prevalence and Age-of-Onset Distributions of DSM-IV Disorders in the National Comorbidity Survey Replication. *Archives of General Psychiatry.* 2005;62(6):593-602.
34. Copeland W, Shanahan L, Costello EJ, Angold A. Cumulative prevalence of psychiatric disorders by young adulthood: a prospective cohort analysis from the Great Smoky Mountains Study. *J Am Acad Child Adolesc Psychiatry.* 2011;50(3):252-261.
35. Farmer RF, Kosty DB, Seeley JR, Olino TM, Lewinsohn PM. Aggregation of lifetime Axis I psychiatric disorders through age 30: incidence, predictors, and associated psychosocial outcomes. *J Abnorm Psychol.* 2013;122(2):573-586.
36. Angst J, Paksarian D, Cui L, et al. The epidemiology of common mental disorders from age 20 to 50: results from the prospective Zurich cohort Study. *Epidemiol Psychiatr Sci.* 2016;25(1):24-32.
37. Wittchen HU. Reliability and validity studies of the WHO--Composite International Diagnostic Interview (CIDI): a critical review. *J Psychiatr Res.* 1994;28(1):57-84.

© 2020 Caspi A et al. *JAMA Network Open.*
